# *Bifidobacterium longum* P77 and *Lactiplantibacillus plantarum* P72 and Their Mix—Live or Heat-Treated—Mitigate Sleeplessness and Depression in Mice: Involvement of Serotonergic and GABAergic Systems

**DOI:** 10.3390/cells14191547

**Published:** 2025-10-03

**Authors:** Ji-Su Baek, Xiaoyang Ma, Hee-Seo Park, Dong-Yun Lee, Dong-Hyun Kim

**Affiliations:** College of Pharmacy, Kyung Hee University, Dongdaemun-gu, Seoul 02447, Republic of Korea; jisoo500@khu.ac.kr (J.-S.B.); xiaoyangma12@khu.ac.kr (X.M.); xlvksl1997@khu.ac.kr (H.-S.P.); dongyun8246@khu.ac.kr (D.-Y.L.)

**Keywords:** *Lactiplantibacillus plantarum* P72, *Bifidobacterium longum* P77, depression, sleeplessness, serotonin, GABA

## Abstract

Sleeplessness (insomnia) is a significant symptom associated with stress-induced depression/anxiety. In the present study, we selected *Bifidobacterium longum* P77, which increased serotonin production in corticosterone-stimulated SH-SY5Y cells, from the fecal bacteria collection of healthy volunteers and examined the effects of *B. longum* on depression, anxiety, and sleeplessness induced by immobilization stress or by transplantation of cultured fecal microbiota (cFM) from patients with depression. Orally administered *B. longum* P77 decreased depression/anxiety- and sleeplessness-like behaviors in immobilization stress-exposed mice. *B. longum* P77 reduced immobilization stress-induced corticosterone, tumor necrosis factor (TNF)-α, and interleukin (IL)-6 expression and the cell population of nuclear factor kappa-light-chain-enhancer of activated B cells (NF-κB)^+^ in the prefrontal cortex, while the expression levels of immobilization stress-suppressed IL-10, γ-aminobutyric acid (GABA), its receptor GABA_A_Rα1, serotonin, and its receptor 5-HT_1A_R increased. *B. longum* P77 also alleviated immobilization stress-induced colitis: it decreased TNF-α and IL-6 expression and increased IL-10 expression in the colon. Furthermore, *B. longum* P77, *Lactiplantibacillus plantarum* P72, and their combination decreased cFM- or immobilization stress-induced depression-, anxiety-, and sleeplessness-like behaviors. They also decreased cFM-induced, corticosterone, TNF-α, and IL-6 expression levels in the prefrontal cortex and colon, while increasing cFM- or immobilization stress-suppressed GABA, GABA_A_Rα1, serotonin, and 5-HT_1A_R expression levels in the prefrontal cortex. In particular, the combination of *B. longum* P77 and *L. plantarum* P72 (P7277) additively or synergistically alleviated depression-, anxiety-, and sleeplessness-like behaviors, along with their associated biomarkers. Heat-killed P7277 also alleviated immobilization stress-induced depression/anxiety- and sleeplessness-like symptoms. These results imply that *L. plantarum* P72 and/or *B. longum* P77 can mitigate depression/anxiety and sleeplessness by upregulating GABAergic and serotonergic systems, along with the suppression of NF-κB activation.

## 1. Introduction

Long-term exposure to stress induces the secretion of adrenal hormones such as corticosterone, aldosterone, and adrenaline as well as proinflammatory cytokines such as tumor necrosis factor (TNF)-α, resulting in anxiety and depression [1,2]. Approximately 90% of patients with depression and anxiety, which are closely associated with decreased serotonin (5-HT) and γ-aminobutyric acid (GABA) levels and elevated interleukin (IL)-6 levels [3,4,5], suffer from sleeplessness [6].

Gut microbiota communicates bidirectionally with the brain through the gut–brain axis [7,8]. Stress-induced gut dysbiosis can contribute to gut inflammation as well as psychiatric disorder, including depression and sleeplessness [9,10,11]. Many probiotics, including *lactobacilli* and *bifidobacteria*, modulate immune responses such as inflammation and hypersensitivity and alleviate psychiatric disorders such as depression/anxiety and sleeplessness [12,13,14]. *Lactiplantibacillus plantarum* D-9, *Bifidobacterium longum* CCFM687, and *B. longum* 1714 alleviate stress-induced depression-like behavior in mice [10,15,16]. *Lactobacillus rhamnosus* GMNL-74 suppresses depression in ampicillin-exposed mice [17]. *Bifidobacterium breve* CCFM1025 improves sleep disorder in stress-exposed mice [18]. *L. plantarum* JYLP-326 simultaneously mitigates depression and sleep disorders in stress-exposed mice [18,19]. A mix of *Lactobacillus reuteri* NK33 and *Bifidobacterium adolescentis* NK98 (NVP1704) mitigates depression/anxiety- and sleeplessness like behaviors in stress-exposed mice [20]. *L. plantarum* P8 alleviates anxiety in stressed adults [21]. *L. plantarum* PS128 improves depression-related symptom in volunteers with self-reported insomnia [22]. *B. breve* CCFM1025 attenuates major depression disorder in volunteers [23]. NVP1704 mitigates depression- and sleeplessness-related symptoms in healthy volunteers with depression and insomnia symptoms [24]. *L. plantarum* JYLP-326 also alleviates depression and insomnia in test-anxious college students [19]. *B. breve* CCFM1025 improves sleep quality in healthy volunteers with stress-induced insomnia [25]. A mix of *Lactiplantibacillus plantarum* P72 and hempseed oil also synergistically mitigates immobilization stress-induced or cultured fecal microbiota of patients with depression (cFM)-induced depression/anxiety and sleeplessness in mice [26]. Nevertheless, the action mechanism by which probiotics alleviate sleeplessness associated with depression and anxiety remains unclear.

Therefore, we isolated *Bifidobacterium longum* P77 from the bacteria collection isolated from healthy feces and examined the effects of *B. longum* P77 and a combination of *L. plantarum* P72 and *B. longum* P77 (P7277) on immobilization stress- or cFM-induced depression/anxiety and sleeplessness in mice. Additionally, the effect of heat-killed P7277 (hP7277) on depression/anxiety and sleeplessness was investigated in immobilization stress-exposed mice.

## 2. Materials and Methods

### 2.1. Culture of B. longum P77 and L. plantarum P72

*B. longum* P77 (KCCM13446P) and *L. plantarum* P72 (KCCM13445P), which were provided by PBLbiolab (Seoul, Korea), were cultured in general media for probiotics such as MRS broth, centrifuged, and freeze-dried, as previously reported [26]. The number of their viable cells was quantified by using a commonly used method such as the culture in the agar plate. Based on the number of live cells, *L. plantarum* P72 and *B. longum* P77 were mixed at a ratio of 4:1. This (4:1) mixture is named P7277. Heat-killed P7277 (hP7277) was prepared by (4:1) mixing *L. plantarum* P72 and *B. longum* P77 heat-killed at 75 °C for 20 min. hP7277 did not grow on the MRS broth. However, there were no observable external differences between P7277 (live) and hP7277 (heat-killed) when examined visually, under a microscope, and through 16S rRNA gene analysis.

### 2.2. Culture of the SH-SY5Y Human Neuroblastoma Cell Line

The SH-SY5Y human neuroblastoma cell line, derived from a bone marrow biopsy of a neuroblastoma patient and widely used as a model for neuronal differentiation and neurodegenerative disease research, was cultured at 37 °C in Dulbecco’s Modified Eagle Medium containing 5% fetal bovine serum (FBS) and 1% antibiotic-antimycotic. Cells (1 × 10^6^ cells/mL) were maintained in a humidified incubator with 5% CO_2_ and 95% air. For experimental treatment, cells were exposed to 300 μM corticosterone (Sigma-Aldrich, Burlington, MA, USA), either alone or in combination with probiotics (1 × 10^5^ colony-forming unit [CFU]/mL), for 24 h. After treatment, culture media were collected, and serotonin concentrations in the supernatants were determined using a commercially available serotonin quantification kit (DLD Diagnostika GmbH, Hamburg, Germany), following the instructions provided by the manufacturer.

### 2.3. Animals

Male C57BL/6 mice (6 weeks old, 18–20 g) were purchased from Koatech (Pyeongtaek-shi, Republic of Korea). Animals were housed in plastic cages under controlled environmental conditions (temperature: 20–22 °C; humidity: 50 ± 10%; 12 h light/dark cycle) and allowed to acclimate for one week prior to experimentation. During the study period, mice had free access to standard laboratory chow and water. All animal procedures were reviewed and approved by the Institutional Animal Care and Use Committee of the University (IACC: KHUASP(SE)_23545) and carried out in accordance with the University’s guidelines and ARRIVE guideline [27] and ethical standards for the care and use of laboratory animals.

### 2.4. Preparation of Mice with Depression/Anxiety and Sleeplessness and Treatment with Test Agents

Mice with depression/anxiety and sleeplessness were prepared by exposing them to immobilization stress or oral gavaging cFM of patients with depression (2 × 10^8^ CFU/0.1 mL/mouse), as previously reported [20].

First, to investigate the effects of *B. longum* P77 and diphenhydramine against depression/anxiety and sleeplessness, mice were divided into four groups: normal control (NC), immobilization stress-exposed (IM), immobilization stress/*B. longum* P77-treated (P77), and immobilization stress/diphenhydramine-treated (DH) groups. Each consisted of eight mice. Except for NC, all groups were subjected to immobilization stress daily for 5 days, as previously reported [20]. Thereafter (24 h after the final stress exposure), test agents (IM, vehicle alone; P77, 1 × 10^9^ CFU/mouse of *B. longum* P77; DH, 20 mg/kg diphenhydramine) were administered once daily for 7 days. NC received saline instead of test agents.

Second, mice were divided into five groups: NC, cFM-exposed (FM), cFM/*L. plantarum* P72-treated (P72), cFM/*B. longum* P77-treated (P77), and cFM/P7277-treated (P72P77) groups. Each group consisted of eight mice. cFM (1 × 10^9^ CFU/mouse of the fecal microbiota culture suspended in 0.1 mL saline) was administered orally to all groups except NC for 5 days, as previously described [28]. Twenty-four h after the final cFM treatment, test agents (FM, vehicle; P72, 1 × 10^9^ CFU/mouse of *L. plantarum* P72; P77, 1 × 10^9^ CFU/mouse of *B. longum* P77; P72P77, 1 × 10^9^ CFU/mouse of P7277) were orally administered once daily for 7 days. NC received saline.

Third, mice were divided into five groups: NC, immobilization stress-treated (IM), immobilization stress/*L. plantarum* P72-treated (P72), immobilization stress/*B. longum* P77-treated (P77), and immobilization stress/P7277-treated (P72P77) groups. Each group consisted of eight mice. Mice, except NC, were exposed to immobilization stress daily for 5 days, as previously reported [29]. Tested agents (IM, vehicle alone; P72, 1 × 10^9^ CFU/mouse of *L. plantarum* P72; P77, 1 × 10^9^ CFU/mouse of *B. longum* P77; P72P77, 1 × 10^9^ CFU/mouse of P7277) were orally administered once daily for 7 days from 24 h after the final exposure to immobilization stress. NC was orally gavaged with saline instead of test agents.

Fourth, mice were divided into three groups: NC, immobilization stress-exposed (IM), and immobilization stress/hP7277-treated (P72P77) groups. Each group consisted of eight mice. Mice, except NC, were exposed to immobilization stress once daily for 5 days [29]. Tested agents (IM, vehicle alone; hP72P77, 1 × 10^9^ CFU/mouse of hP7277) were orally administered once daily for 7 days from 24 h after the final exposure to immobilization stress. NC was orally gavaged with saline instead of test agents.

Depression-, anxiety-, and sleeplessness-like behaviors were assessed using multiple behavioral tests, as previously reported [30]. In the open field test, total distance moved, distance traveled in the center area, and time spent in the center area were recorded. In the elevated plus maze test, time spent in the open arms and entry number into the open arms were measured. In the tail suspension test, the immobility time was recorded. Thereafter, sleep latency time and sleep duration were measured (between 1:00 and 5:00 pm) after the exposure to isoflurane in the chamber or the intraperitoneal injection of pentobarbital sodium (40 mg/kg) [26,31]. Detailed protocols are described in Supplementary Methods.

For biomarker analysis, mice were euthanized by CO_2_ exposure in a closed chamber followed by cervical dislocation. Brain and colon tissues were promptly harvested and stored at −80 °C until further analysis. For immunofluorescence procedures, mice underwent transcardial perfusion with 4% paraformaldehyde, followed by cryoprotection in a 30% sucrose solution. Tissue samples were then processed for immunofluorescence staining according to previously established protocols [29].

### 2.5. Enzyme-Linked Immunosorbent Assay (ELISA) and Quantitative Polymerase Chain Reaction (qPCR)

Cytokine, serotonin, and GABA levels in the prefrontal cortex and colon were assessed using ELISA kits. Serotonin 1A receptor (5-HT_1A_R), 5-HT_1B_R, GABA type A receptor subunit alpha1 (GABA_A_Rα1), GABA type A receptor subunit alpha2 (GABA_A_Rα2), and 18S rRNA gene levels were accessed, using qPCR, as previously reported [20]. Primers are indicated in Supplementary Table S1. Detailed protocols are described in Supplementary Methods.

### 2.6. Whole Genome Analysis

The whole genome sequence for *B. longum P77* was analyzed, as previously reported [26].

### 2.7. Statistical Analysis

Experimental data are indicated as mean ± standard deviation (SD) and accessed using GraphPad Prism 9. The significance difference was analyzed using one-way ANOVA with Tukey multiple comparisons test (*p* < 0.05). The statistical data are indicated in Supplementary Table S2.

## 3. Results

### 3.1. B. longum P77 Induced the Release of Serotonin in Corticosterone-Exposed SH-SY5Y Cells

**Figure 1:**
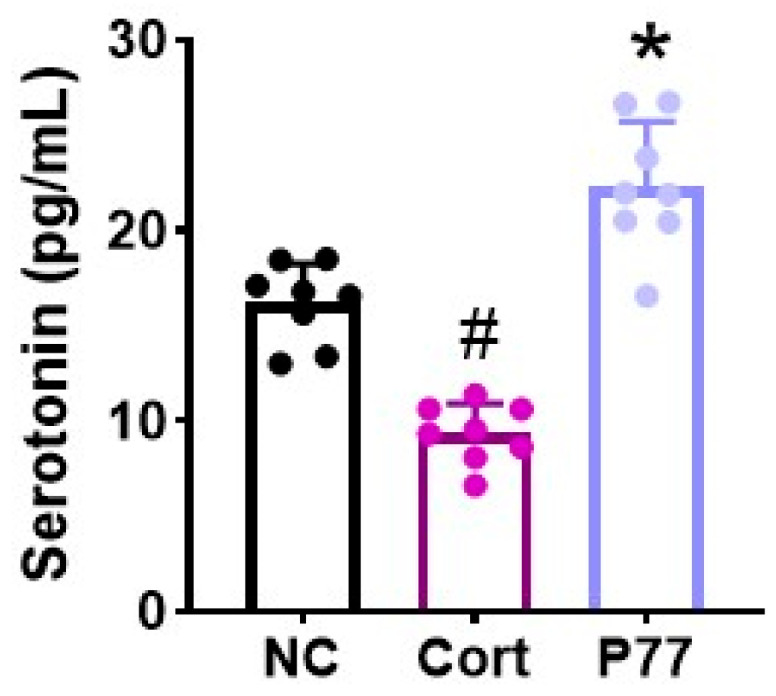
Effect of *B. longum* P77 on the serotonin production in corticosterone (Cort)-stimulated SH-SY5Y cells. *n* = 8. ^#^ *p* < 0.05 vs. NC. * *p* < 0.05 vs. Cort.

To search serotonin production-inducing gut bifidobacteria, we measured the effects of bifidobacteria, which were selected from the bacteria collection of healthy human feces, on the release of serotonin in corticosterone-exposed SH-SY5Y cells. Among the tested strains, P77 markedly increased serotonin production (Figure 1). P77 was named *B. longum* P77, based on Gram staining, API kit, and 16S rRNA gene sequencing. The pseudogenome of *B. longum* P77 was 2,407,312 base pairs (contig 1) with a GC content of 60% (Supplementary Figure S1). The total number of CDS was 2014. The tRNA gene number was 62. The rRNA gene number was 13. The whole genome sequence of *B. longum* P77 exhibited the highest phylogenetic similarity to *B. longum* JCM1217 (98.6%), using OrthoANI.

### 3.2. B. longum P77 Attenuated Depression/Anxiety- and Sleeplessness-Like Symptoms in Immobilization Stress-Exposed Mice

**Figure 2:**
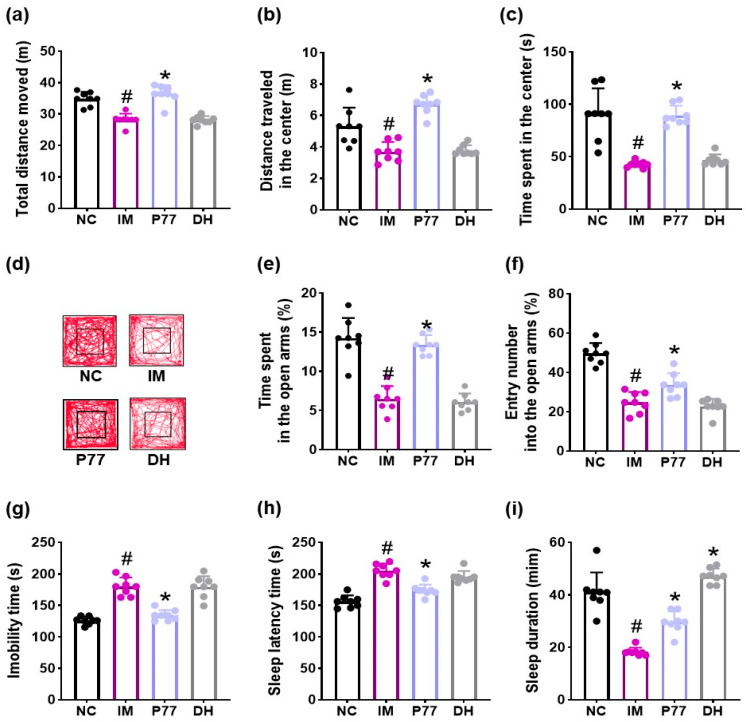
Effects of *B. longum* P77 and diphenhydramine on immobilization stress-induced depression/anxiety- and sleeplessness-like behaviors in mice. Effects on total distance moved (**a**), distance traveled in the center (**b**), time spent in the center (**c**), and track path (**d**) in the open field test. Effects on time spent in the open arms (**e**) and entry number into the open arms (**f**) in the elevated plus maze test and immobility time in the tail suspension test (**g**). Effects on sleep latency time (**h**) and sleep duration (**i**). Test agents (IM, vehicle; P77, 1 × 10^9^ CFU/mouse/day of *B. longum* P77; DH, 20 mg/kg of diphenhydramine) were treated in immobilization stress/pentobarbital-exposed mice. NC was treated with saline in immobilization stress-untreated mice. *n* = 8. ^#^ *p* < 0.05 vs. NC. * *p* < 0.05 vs. IM.

To understand whether a serotonin production-enhancing probiotic *B. longum* P77 could alleviate psychiatric disorders, we examined their antidepressant effects in mice with immobilization stress-induced depression- and anxiety-like symptoms (Figure 2). Exposure to immobilization stress caused depression/anxiety-like behaviors: it reduced total distance moved, distance traveled in the center, and time spent in the center in the open field test and time spent in the open arms and entry number into the open arms in the elevated plus maze test to 81.0% (F_3.28_ = 33.92, *p* < 0.001), 69.5% (F_3.28_ = 28.94, *p* < 0.001), 46.8% (F_3.28_ = 31.16, *p* < 0.001), 45.6% (F_3.28_ = 18.72, *p* < 0.001), and 49.9% (F_3.28_ = 44.67, *p* < 0.001) of those observed in NC, respectively, and increased immobility time in the tail suspension test to 143.1% (F_3.28_ = 46.20, *p* < 0.001) of that observed in NC.

In contrast, oral administration of *B. longum* P77 alleviated immobilization stress-induced depression- and anxiety-like behaviors. It recovered immobilization stress-suppressed total distance moved, distance traveled in the center, time spent in the center, time spent in the open arms, and entry number into the open arms to 104.5%, 126.5%, 98.1%, 94.0%, and 67.9% of those observed in NC, respectively. *B. longum* P77 reduced immobilization stress-induced immobility time to 106.7% of that observed in NC.

Exposure to immobilization stress caused sleeplessness in mice: it increased pentobarbital-induced sleep latency time to 131.3% (F_3,28_ = 39.31, *p* < 0.001) of that observed in NC and decreased pentobarbital-induced sleep duration to 44.5% (F_3,28_ = 63.24, *p* < 0.001) of that observed in NC. However, *B. longum* P77 partially recovered sleeplessness in mice. It recovered immobilization stress-increased sleep latency time to 111.4% of that observed in NC and immobilization stress-decreased sleep duration to 72.2% of that observed in NC. Its potency was comparable to that of diphenhydramine.

**Figure 3:**
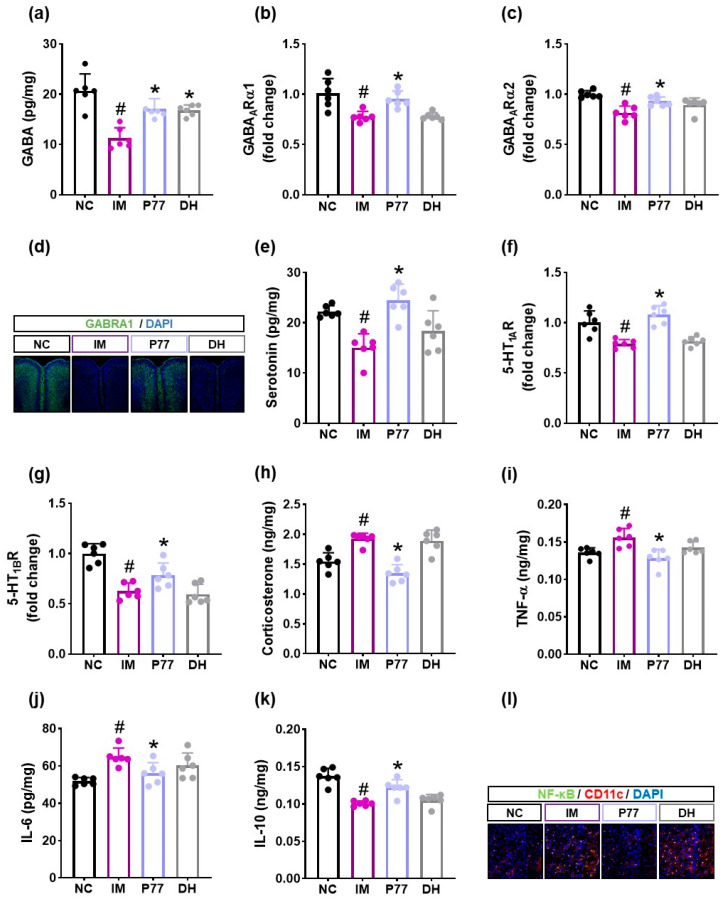
Effects of *B. longum* P77 and diphenhydramine on the expression of depression/anxiety- and sleeplessness-related biomarkers in the prefrontal cortex of mice exposed to immobilization stress. Effects on GABA (**a**), GABA_A_Rα1 (**b**), and GABA_A_Rα2 (**c**) expression levels and GABA_A_Rα1^+^ cell population (**d**). Effects on serotonin (**e**), 5-HT_1A_R (**f**), and 5-HT_1B_R (**g**) expression levels. Effects on corticosterone (**h**), TNF-α (**i**), IL-6 (**j**) and IL-10 (**k**) expression and NF-κB^+^CD11c^+^ cell population (**l**). Test agents (IM, vehicle; P77, 1 × 10^9^ CFU/mouse/day of *B. longum* P77; DH, 20 mg/kg of diphenhydramine) were treated in immobilization stress/pentobarbital-exposed mice. NC was treated with saline in immobilization stress-untreated mice. *n* = 8. ^#^ *p* < 0.05 vs. NC. * *p* < 0.05 vs. IM.

Exposure to immobilization stress decreased the expression levels of GABA, serotonin, their receptors (GABA_A_Rα1, GABA_A_Rα2, 5-HT_1A_R, 5-HT_1B_R), and IL-10 and increased the expression levels of corticosterone, TNF-α, and IL-6 and population of nuclear factor kappa-light-chain-enhancer of activated B cells (NF-κB)-positive cells in the prefrontal cortex (Figure 3, Supplementary Figure S2). However, treatment with *B. longum* P77 increased immobilization stress-downregulated GABA, serotonin, their receptors, GABA_A_Rα1, GABA_A_Rα2, 5-HT_1A_R, 5-HT_1B_R, and IL-10 expression and the GABA_A_Rα1^+^ cell population in the prefrontal cortex while decreasing immobilization stress-induced corticosterone levels. *B. longum* P77 decreased immobilization stress-induced TNF-α and IL-6 expression and the NF-κB^+^CD11c^+^ cell population and increased immobilization stress-suppressed IL-10 expression in the prefrontal cortex.

**Figure 4:**
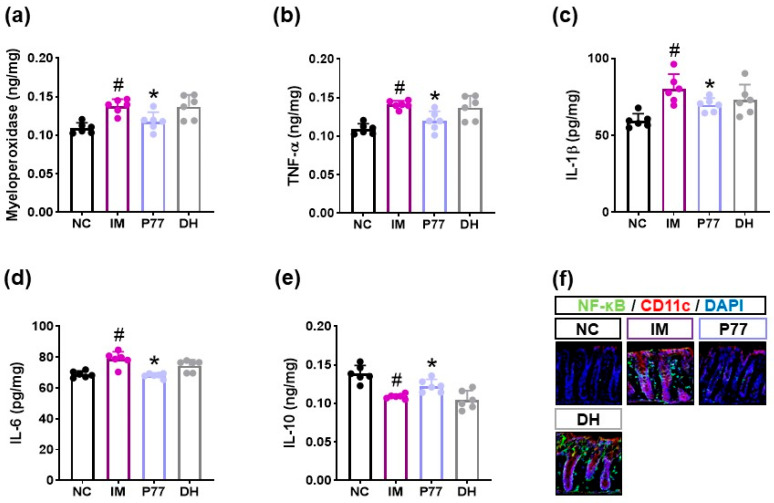
Effects of *B. longum* P77 and diphenhydramine on immobilization stress-induced colitis in mice. Effects on myeloperoxidase (**a**), TNF-α (**b**), IL-1β (**c**), IL-6 (**d**), and IL-10 expression (**e**) and NF-κB^+^CD11c^+^ cell population (**f**) in the colon. Test agents (IM, vehicle; P77, 1 × 10^9^ CFU/mouse/day of *B. longum* P77; DH, 20 mg/kg of diphenhydramine) were treated in immobilization stress/pentobarbital-exposed mice. NC was treated with saline in immobilization stress-untreated mice. *n* = 8. ^#^ *p* < 0.05 vs. NC. * *p* < 0.05 vs. IM.

Gut dysbiosis-induced gut inflammation is closely involved in the occurrence of psychiatric disorder, including depression [11]. Therefore, we examined whether immobilization stress could cause colitis, a symptom of depression, and *B. longum* P77 could alleviate immobilization stress-induced colitis. Exposure to immobilization stress caused colitis: it increased myeloperoxidase, TNF-α, IL-1β, and IL-6 expression and decreased IL-10 expression in the colon (Figure 4, Supplementary Figure S3). However, oral administration of *B. longum* P77 significantly decreased immobilization stress-induced myeloperoxidase, TNF-α, IL-1β, and IL-6 expression and the NF-κB^+^CD11c^+^ cell population.

### 3.3. B. longum P77, L. plantarum P72, and P7277 Alleviated cFM Transplantation-Induced Depression/Anxiety- and Sleeplessness-Like Behaviors and Gut Inflammation in Mice

**Figure 5:**
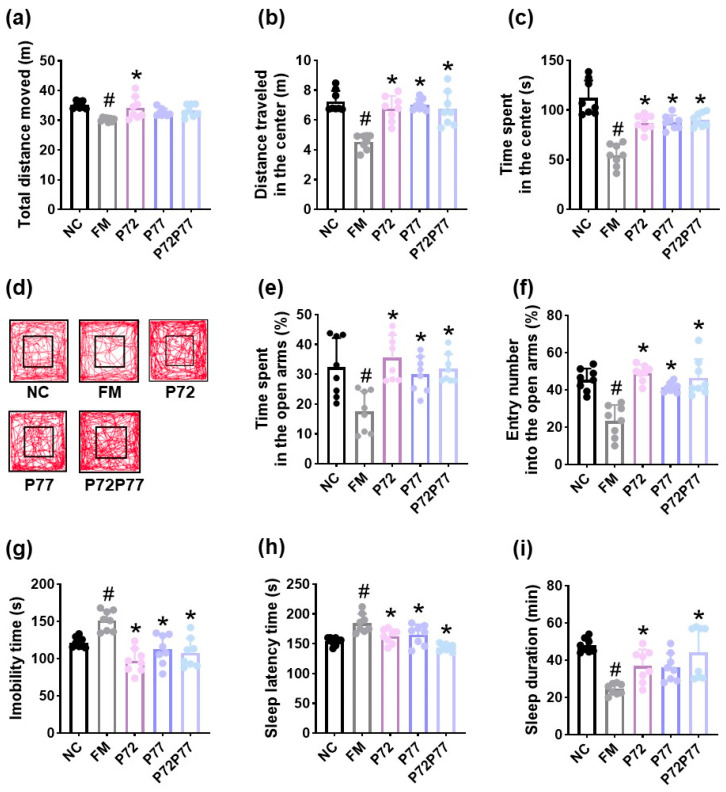
Effects of *L. plantarum* P72, *B. longum* P77, and P7277 on cFM-induced depression/anxiety- and sleeplessness-like behaviors in mice. Effects on total distance moved (**a**), distance traveled in the center (**b**), time spent in the center (**c**), and track path (**d**) in the open field test. Effects on time spent in the open arms (**e**) and entry number into the open arms (**f**) in the elevated plus maze test and immobility time in the tail suspension test (**g**). Effects on sleep latency time (**h**) and sleep duration (**i**). Test agents (FM, vehicle; P72, 1 × 10^9^ CFU/mouse/day of *L. plantarum* P72; P77, 1 × 10^9^ CFU/mouse/day of *B. longum* P77; P72P77, 1 × 10^9^ CFU/mouse/day of P7277) were treated in cFM/pentobarbital-exposed mice. NC was treated with saline in cFM-untreated mice. *n* = 8. ^#^ *p* < 0.05 vs. NC. * *p* < 0.05 vs. FM.

To determine whether the depression-, anxiety- and sleeplessness-alleviating activity of *B. longum* P77 could be synergistically enhanced by *L. plantarum* P72, we examined the effects of *B. longum* P77, *L. plantarum* P72, and P7277 on cFM transplantation-induced depression/anxiety- and sleeplessness-like symptoms in mice (Figure 5).

cFM transplantation decreased total distance moved, distance traveled in the center, time spent in the center, time spent in the open arms, and entry number into the open arms to 86.1% (F_4.35_ = 6.418, *p* < 0.001), 62.6% (F_4.35_ = 16.37, *p* < 0.001), 48.6% (F_4.35_ = 28.49, *p* < 0.001), 54.0% (F_4.35_ = 7.509, *p* = 0.0002), and 51.4% (F_4.35_ = 17.94, *p* < 0.001) of those observed in NC, respectively, and increased immobility time to 124.2% (F_4.35_ = 13.14, *p* < 0.001) of that observed in NC. However, orally administered *L. plantarum* P72, *B. longum* P77, and P7277 significantly increased cFM-decreased distance traveled in the center to 93.4%, 97.0%, and 92.8% of that observed in NC, respectively, time spent in the center to 77.7%, 78.2% and 80.5% of that observed in NC, respectively, and time spent in the open arms to 110.0%, 92.6%, and 98.6% of that observed in NC, respectively, and entry number into the open arms to109.2%, 91.5%, and 98.2% of that observed in NC, respectively, and decreased cFM-increased immobility time to 79.9%, 92.6%, and 88.6% of that observed in NC, respectively, while FM-decreased total distance moved was significantly increased by treatment with *L. plantarum* P72 alone.

cFM transplantation caused sleeplessness-like behavior: it increased pentobarbital-induced sleep latency time to 119.5% (F_4.35_ = 13.14, *p* < 0.001) of that observed in NC and decreased pentobarbital-induced sleep duration to 50.9% (F_4.35_ = 9.33, *p* ≤ 0.001) of that observed in NC. However, oral administration of *L. plantarum* P72, *B. longum* P77, and P7277 significantly decreased cFM-increased sleep latency time to 105.4%, 107.3%, and 93.1% of that observed in NC, respectively, and increased cFM-decreased sleep duration to 77.2%, 75.5%, and 92.4% of that observed in NC, respectively.

**Figure 6:**
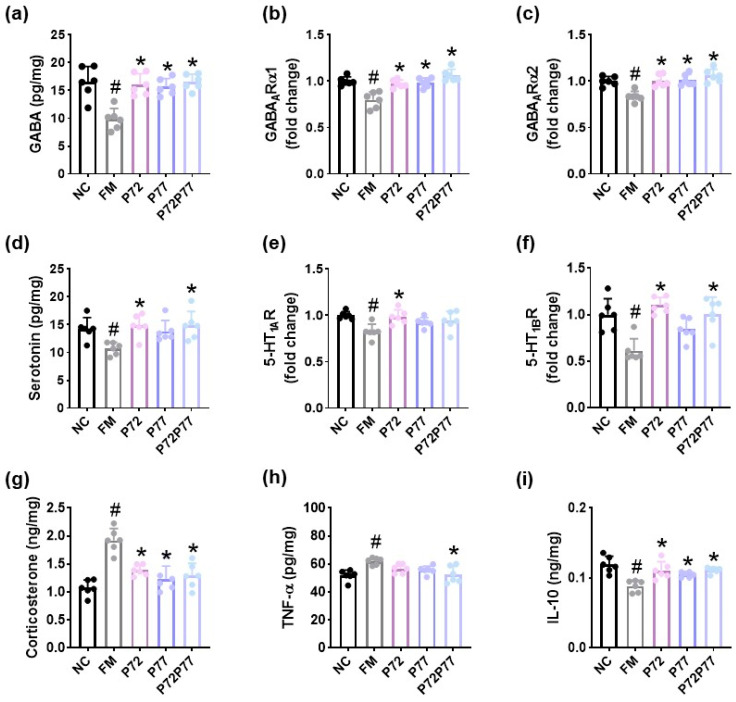
Effects of *L. plantarum* P72, *B. longum* P77, and P7277 on the expression of depression/anxiety- and sleeplessness-related biomarkers in the prefrontal cortex of mice exposed to cFM. Effects on GABA (**a**), GABA_A_Rα1 (**b**), and GABA_A_Rα2 (**c**) expression levels. Effects on serotonin (**d**), 5-HT_1A_R (**e**), and 5-HT_1B_R (**f**) expression levels. Effects on corticosterone (**g**), TNF-α (**h**) and IL-10 expression (**i**). Test agents (FM, vehicle; P72, 1 × 10^9^ CFU/mouse/day of *L. plantarum* P72; P77, 1 × 10^9^ CFU/mouse/day of *B. longum* P77; P72P77, 1 × 10^9^ CFU/mouse/day of P7277) were treated in cFM/pentobarbital-exposed mice. NC was treated with saline in cFM-untreated mice. *n* = 8. ^#^ *p* < 0.05 vs. NC. * *p* < 0.05 vs. FM.

cFM transplantation decreased serotonin, GABA, GABA_A_Rα1, GABA_A_Rα2, 5-HT_1A_R, 5-HT_1B_R, and IL-10 expression and increased corticosterone and TNF-α expression in the prefrontal cortex (Figure 6). However, oral administration of *L. plantarum* P72, *B. longum* P77, and P7277 significantly increased cFM-suppressed GABA, serotonin, GABA_A_Rα1, GABA_A_Rα2, and IL-10 expression and decreased cFM-induced corticosterone levels. They weakly increased cFM-suppressed 5-HT_1A_R and 5-HT_1B_R expression and decreased cFM-induced TNF-α expression.

**Figure 7:**
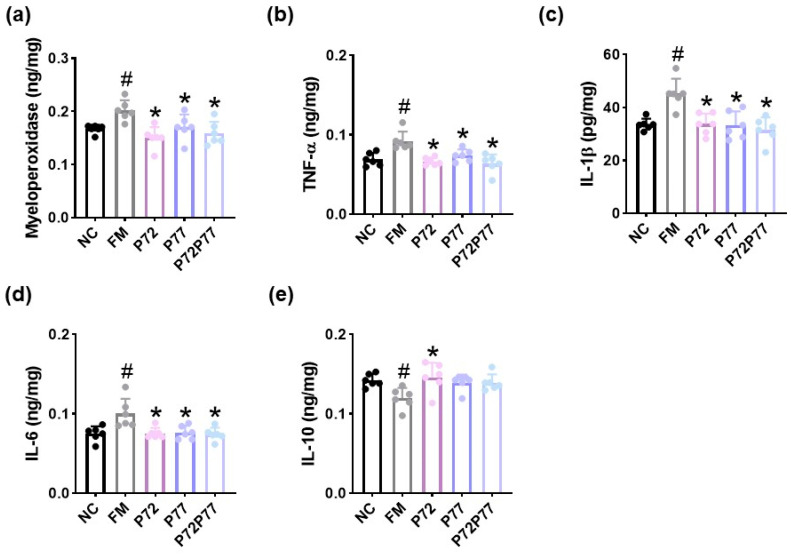
Effects of *L. plantarum* P72, *B. longum* P77, and P7277 on cFM-induced colitis in mice. Effects on myeloperoxidase (**a**), TNF-α (**b**), IL-1β (**c**), IL-6 (**d**), and IL-10 expression (**e**) in the colon. Test agents (FM, vehicle; P72, 1 × 10^9^ CFU/mouse/day of *L. plantarum* P72; P77, 1 × 10^9^ CFU/mouse/day of *B. longum* P77; P72P77, 1 × 10^9^ CFU/mouse/day of P7277) were treated in cFM-exposed mice. NC was treated with saline in cFM/pentobarbital-untreated mice. *n* = 8. ^#^ *p* < 0.05 vs. NC. * *p* < 0.05 vs. FM.

Next, we examined whether cFM transplantation could cause colitis, a symptom of depression, and *L. plantarum* P72, *B. longum* P77, and P7277 could alleviate immobilization stress-induced colitis. cFM transplantation also caused colitis in mice: it increased myeloperoxidase, TNF-α, IL-1β, and IL-6 expression and decreased IL-10 expression in the colon (Figure 7). However, oral administration of *L. plantarum* P72, *B. longum* P77, or P7277 significantly suppressed cFM-induced myeloperoxidase, TNF-α, IL-1β, and IL-6 expression. *L. plantarum* P72 alone significantly increased FM-suppressed IL-10 expression.

### 3.4. L. plantarum P72, B. longum P77, and P7277 Alleviated Immobilization Stress-Induced Depression/Anxiety- and Sleeplessness-Like Behavior and Systemic Inflammation in Mice

**Figure 8:**
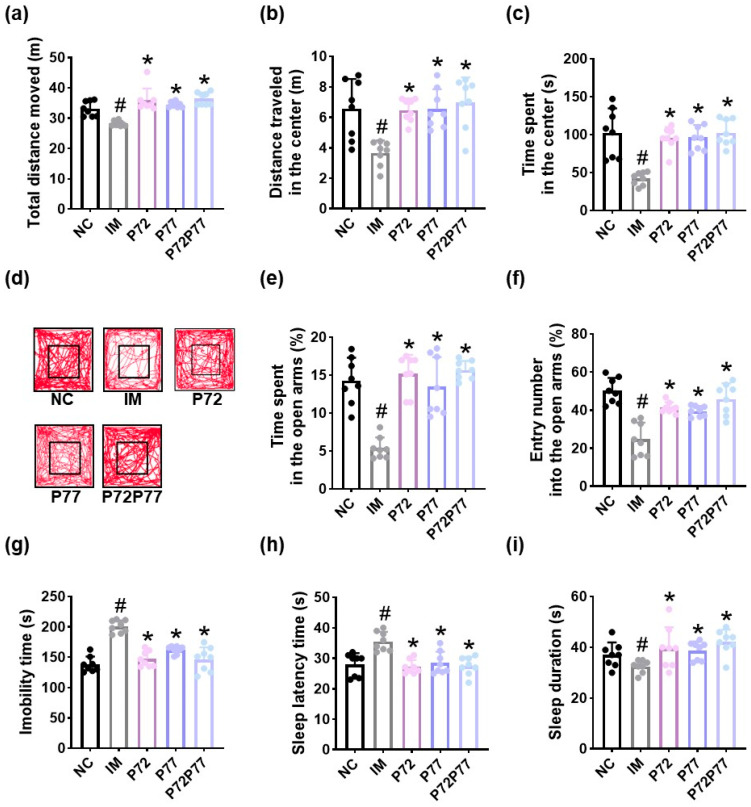
Effects of *L. plantarum* P72, *B. longum* P77, and P7277 on immobilization stress-induced depression/anxiety- and sleeplessness-like behaviors in mice. Effects on total distance moved (**a**), distance traveled in the center (**b**), time spent in the center (**c**), and track path (**d**) in the open field test. Effects on time spent in the open arms (**e**) and entry number into the open arms (**f**) in the elevated plus maze test and immobility time in the tail suspension test (**g**). Effects on sleep latency time (**h**) and sleep duration (**i**). Test agents (IM, vehicle; P72, 1 × 10^9^ CFU/mouse/day of *L. plantarum* P72; P77, 1 × 10^9^ CFU/mouse/day of *B. longum* P77; P72P77, 1 × 10^9^ CFU/mouse/day of P7277) were treated in immobilization stress/isoflurane-exposed mice. NC was treated with saline in immobilization stress-untreated mice. *n* = 8. ^#^ *p* < 0.05 vs. NC. * *p* < 0.05 vs. IM.

Next, we examined the effects of *L. plantarum* P72, *B. longum* P77, and P7277 on immobilization stress-induced depression/anxiety- and sleeplessness-like symptoms in mice (Figure 8). Exposure to immobilization stress significantly decreased total distance moved, distance traveled in the center, time spent in the center, time spent in the open arms, and entry number into the open arms to 85.4% (F_4.35_ = 15.74, *p* < 0.001), 55.7% (F_4.35_ = 7.71, *p* = 0.001), 41.0% (F_4.35_ = 14.03, *p* < 0.001), 37.7% (F_4.35_ = 21.06, *p* < 0.001), and 49.3% (F_4.35_= 18.32, *p* < 0.001) of those observed in NC, respectively. Immobilization stress increased immobility time to 145.2% (F_4.35_ = 28.49, *p* < 0.001) of that observed in NC. However, oral administration of *L. plantarum* P72, *B. longum* P77, and P7277 significantly recovered immobilization stress-suppressed total distance moved to 108.5%, 104.2%, and 110% of that observed in NC, respectively, distance traveled in the center to 98.5%, 99.6%, and 106.1% of that observed in NC, respectively, time spent in the center to 92.7%, 93.7 and 99.7% of that observed in NC, respectively, time spent in the open arms to 106.6%, 94.6%, and 110.0% of that observed in NC, respectively, and entry number into the open arms to 82.3%, 75.9%, and 91.7% of that observed in NC, respectively. They decreased immobility time to 108.5%, 104.2%, and 110.0% of that observed in NC, respectively.

Immobilization stress treatment caused sleeplessness-like behavior: it increased isoflurane-induced sleep latency time to 127.3% (F_4.35_ = 9.67, *p* < 0.01) of that observed in NC and decreased isoflurane-induced sleep duration to 87.8% (F_4.35_ = 3.85, *p* = 0.011) of that observed in NC. However, oral administration of *L. plantarum* P72, *B. longum* P77, or P7277 significantly decreased immobilization stress-induced sleep latency time to 97.2%, 102.0%, and 98.4% of that observed in NC, respectively, and increased immobilization stress-decreased sleep duration to 107.2%, 104.7% and 113.9% of that observed in NC, respectively.

**Figure 9:**
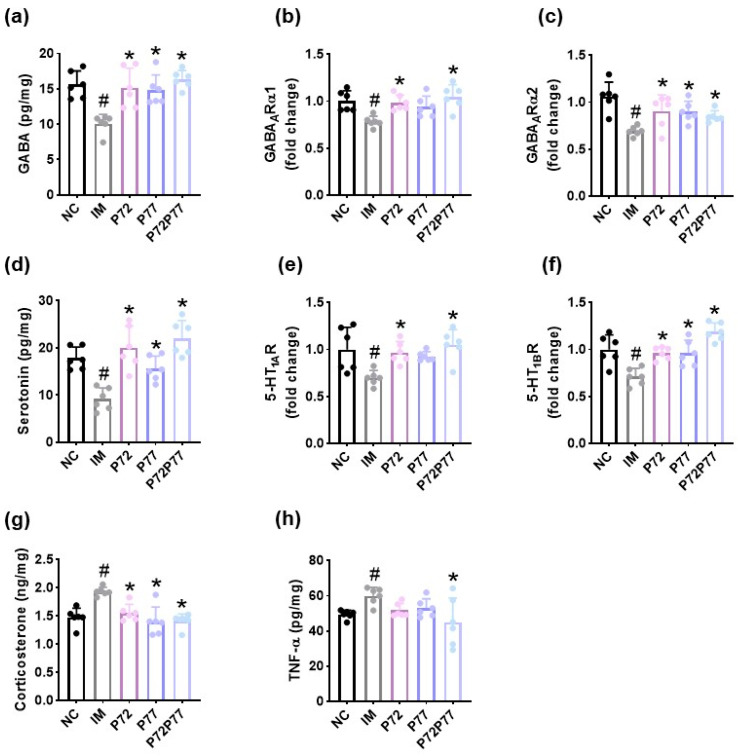
Effects of *L. plantarum* P72, *B. longum* P77, and P7277 on the expression of depression/anxiety- and sleeplessness-related biomarkers in the prefrontal cortex of mice exposed to immobilization stress. Effects on GABA (**a**), GABA_A_Rα1 (**b**), and GABA_A_Rα2 (**c**) expression levels. Effects on serotonin (**d**), 5-HT_1A_R (**e**), and 5-HT_1B_R (**f**) expression levels. Effects on corticosterone (**g**) and TNF-α (**h**) expression. Test agents (IM, vehicle; P72, 1 × 10^9^ CFU/mouse/day of *L. plantarum* P72; P77, 1 × 10^9^ CFU/mouse/day of *B. longum* P77; P72P77, 1 × 10^9^ CFU/mouse/day of P7277) were treated in immobilization stress/isoflurane-exposed mice. NC was treated with saline in immobilization stress-untreated mice. *n* = 8. ^#^ *p* < 0.05 vs. NC. * *p* < 0.05 vs. IM.

Exposure to immobilization stress downregulated GABA, serotonin, and their receptors GABA_A_Rα1, GABA_A_Rα2, 5-HT_1A_R, and 5-HT_1B_R expression and upregulated corticosterone and TNF-α expression in the prefrontal cortex (Figure 9). However, oral administration of *L. plantarum* P72, *B. longum* P77, or P7277 increased immobilization stress-suppressed GABA, serotonin, GABA_A_Rα1 and GABA_A_Rα2, 5-HT_1A_R, and 5-HT_1B_R expression in the prefrontal cortex while decreasing the corticosterone level. *L. plantarum* P72 more potently increased immobilization stress-suppressed GABA_A_Rα1 and 5-HT_1A_R expression than *B. longum* P77. Immobilization stress-induced TNF-α expression was suppressed by P7277 alone.

**Figure 10:**
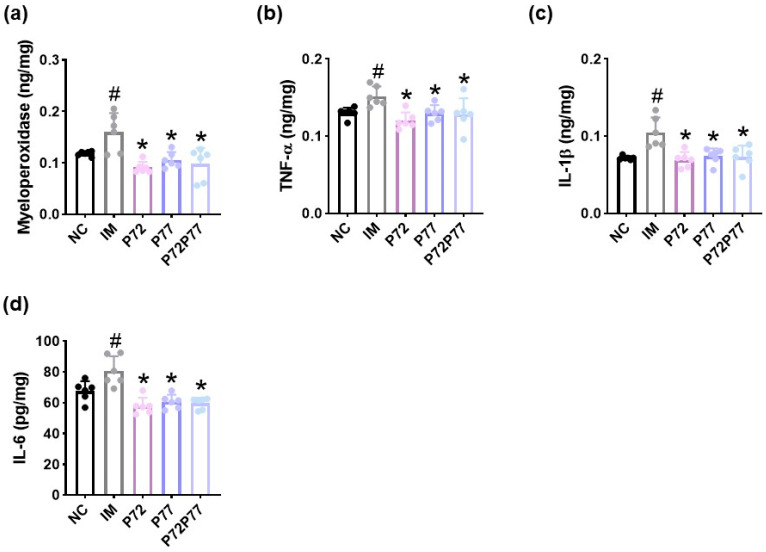
Effects of *L. plantarum* P72, *B. longum* P77, and P7277 on immobilization stress-induced colitis in mice. Effects on myeloperoxidase (**a**), TNF-α (**b**), IL-1β (**c**) and IL-6 (**d**) expression in the colon. Test agents (IM, vehicle; P72, 1 × 10^9^ CFU/mouse/day of *L. plantarum* P72; P77, 1 × 10^9^ CFU/mouse/day of *B. longum* P77; P72P77, 1 × 10^9^ CFU/mouse/day of P7277) were treated in immobilization stress/isoflurane-exposed mice. NC was treated with saline in immobilization stress-untreated mice. *n* = 8. ^#^ *p* < 0.05 vs. NC. * *p* < 0.05 vs. IM.

Next, we examined whether *L. plantarum* P72, *B. longum* P77, and P7277 could alleviate immobilization stress-induced colitis, a symptom of depression, in mice. Exposure to immobilization stress also caused colitis in mice, increasing myeloperoxidase, TNF-α, IL-1β, and IL-6 expression in the colon (Figure 10). However, oral administration of *L. plantarum* P72, *B. longum* P77, or P7277 caused a reduction in immobilization stress-induced myeloperoxidase, TNF-α, IL-1β, and IL-6 expression.

### 3.5. hP7277 Alleviated Immobilization Stress-Induced Depression/Anxiety- and Sleeplessness-Like Behavior and Neuroinflammation in Mice

**Figure 11:**
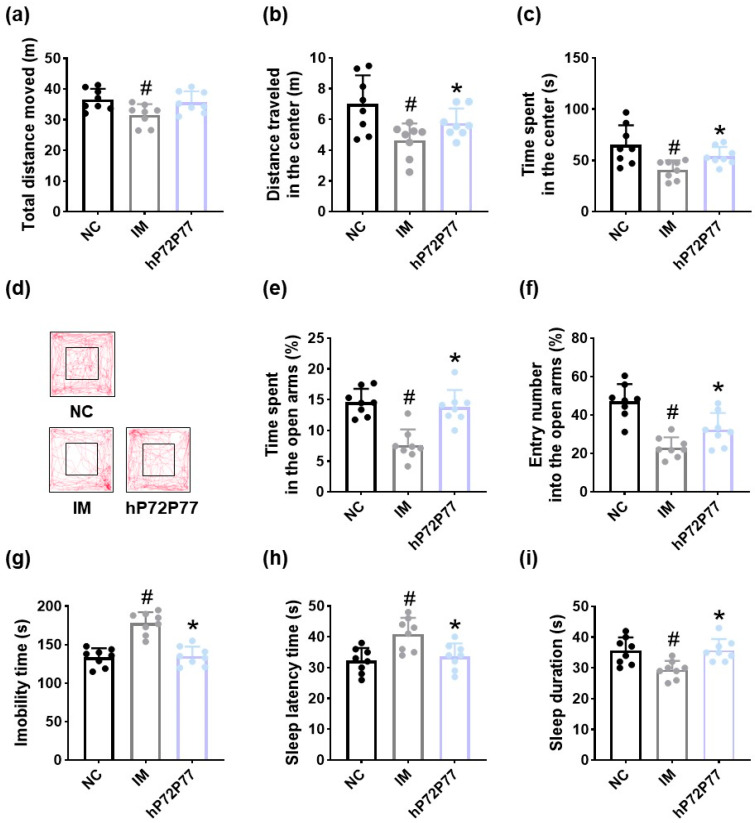
Effect of heat-killed P7277 (hP72P77) on immobilization stress-induced depression/anxiety- and sleeplessness-like behaviors in mice. Effect on total distance moved (**a**), distance traveled in the center (**b**), time spent in the center (**c**), and track path (**d**) in the open field test. Effects on time spent in the open arms (**e**) and entry number into the open arms (**f**) in the elevated plus maze test and immobility time in the tail suspension test (**g**). Effect on sleep latency time (**h**) and sleep duration (**i**). Test agents (IM, vehicle; hP72P7, 1 × 10^9^ CFU/mouse/day of heat-killed *L. plantarum* P72 and *B. longum* P77 [4:1] mix) were treated in immobilization stress/isoflurane-exposed mice. NC was treated with saline in immobilization stress-untreated mice. *n* = 8. ^#^ *p* < 0.05 vs. NC. * *p* < 0.05 vs. IM.

Next, we examined whether heat-killed P7277 (hP7277) could alleviate depression/anxiety- and sleeplessness-like symptoms in immobilization stress-exposed mice (Figure 11). Immobilization stress treatment also significantly caused depression/anxiety- and sleeplessness-like behaviors: it total distance moved, distance traveled in the center, time spent in the center, time spent in the open arms, and entry number into the open arms to 86.3% (F_2,21_ = 4.69, *p* = 0.02), 66.2% (F_2,21_ = 5.98, *p* < 0.001), 62.7% (F_2,21_ = 6.71, *p* < 0.001), 52.3% (F_2,21_ = 18.72, *p* < 0.001), and 49.0% (F_2,21_ = 30.65, *p* < 0.001) of those observed in NC, respectively, and increased immobility time to 133.1% (F_2,21_ = 30.65, *p* < 0.001) of that observed in NC. However, hP7277 recovered immobilization stress-suppressed total distance moved, distance traveled in the center, time spent in the center, time spent in the open arms, and entry number into the open arms to 98.7%, 80.8%, 82.0%, 95.2%, and 68.7% of those observed in NC, respectively. hP7277 decreased immobilization stress-induced immobility time to 101.1% of that observed in NC. Immobilization stress treatment increased isoflurane-induced sleep latency time to 126.7% (F_2,21_ = 8.19, *p* < 0.001) of that observed in NC and decreased isoflurane-induced sleep duration to 82.7% (F_2,21_ = 7.62, *p* < 0.001) of that observed in NC. hP7277 reduced immobilization stress-induced sleep latency time to 104.3% of that observed in NC and increased immobilization stress-decreased sleep duration to 101.0% of NC. However, depression/anxiety- and sleeplessness-like behaviors-ameliorating effect of hP7277 was slightly weaker than that of live P7277: total distance moved, distance traveled in the center, time spent in the center, time spent in the open arms, entry number into the open arms, immobility time, sleep latency time, and sleep duration in mice treated with hP7277 were 89.7%, 76.2%, 82.2%, 86.5%, 74.9%, 91.9, 106.0, and 88.7% of those in mice treated with live P7277, respectively.

**Figure 12:**
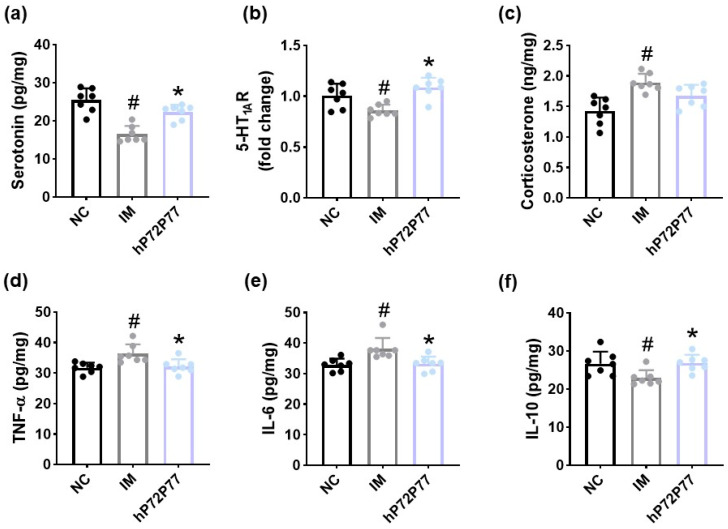
Effect of hP7277 on the expression of depression/anxiety- and sleeplessness-related biomarkers in the prefrontal cortex and colon of mice exposed to immobilization stress. Effect on serotonin (**a**), 5-HT_1A_R (**b**), and corticosterone (**c**) levels in the prefrontal cortex. Effects on TNF-α (**d**), IL-6 (**e**), and IL-10 expression (**f**) in the colon. Test agents (IM, vehicle; hP72P77, 1 × 10^9^ CFU/mouse/day of heat-killed *L. plantarum* P72 and *B. longum* P77 [4:1] mix) were treated in immobilization stress/isoflurane-exposed mice. NC was treated with saline in immobilization stress-untreated mice. n = 8. ^#^ *p* < 0.05 vs. NC. * *p* < 0.05 vs. IM.

hP7277 significantly increased immobilization stress-suppressed serotonin and 5-HT_1A_R expression in the prefrontal cortex and IL-10 expression in the colon, while immobilization stress-induced TNF-α and IL-6 expression in the colon (Figure 12). The immobilization stress-induced corticosterone level in the prefrontal cortex was weakly, but not significantly, decreased.

## 4. Discussion

Stress causes depression and anxiety, which are accompanied by sleeplessness and gut dysbiosis [32,33]. Gut dysbiosis can cause psychiatric disorders including depression/anxiety [11,34] and systemic inflammation [35]. We also found that exposure to immobilization stress induced depression/anxiety and sleeplessness in mice. Furthermore, exposure to immobilization stress suppressed the production of GABA and serotonin and expression of GABA_A_Rα1, GABA_A_Rα2, 5-HT_1A_R, and 5-HT_1B_R in the brain, while TNF-α and IL-6 expression was induced. Immobilization stress also induced TNF-α and IL-6 expression in the colon. The transplantation of depressive patient-derived fecal microbiota also caused depression/anxiety- and sleeplessness-like behaviors in mice. The transplantation suppressed the expression of GABA, serotonin, and their receptors in the brain. Stress including immobilization stress induces gut dysbiosis, as well as systemic inflammation, which reduces serotonin and GABA production in the brain [29,36,37,38]. The transplantation of gut microbiota from volunteers with depression causes depression/anxiety and neuroinflammation in mice [39,40]. These results imply that exposure to immobilization stress can cause depression/anxiety and sleeplessness through the inflammation-involved suppression of serotonin and GABA systems.

Therefore, we selected serotonin production-inducing *B. longum* P77 from the bacterial collection of healthy human feces. The dose of *B. longum* P77 in mouse experiments (1 × 10^9^ CFU/mouse/day) was decided based on the results of animal and in vitro experiments (4 × 10^8^ CFU/mouse/day and 1 × 10^9^ CFU/mouse day in the in vivo experiment and 1 × 10^9^ CFU/mL in the in vitro experiment) previously conducted with *L. plantarum* P72 [26]. Furthermore, *B. longum* P77 (1 × 10^9^ CFU/mouse day) alleviated immobilization stress- or cFM-induced depression/anxiety- and sleeplessness-like behaviors. Furthermore, *B. longum* P77 upregulated immobilization stress- or cFM-suppressed GABA, serotonin, GABA_A_Rα1, GABA_A_Rα2, 5-HT_1A_R, and 5-HT_1B_R expression in the brain. Moreover, *B. longum* P77 downregulated immobilization stress- or cFM-induced TNF-α, IL-6, and NF-κB^+^ cell population in the brain and colon. GABA and benzodiazepines (GABA_A_R agonists) mitigate depression/anxiety and insomnia [41]. 5-HT_1A_R and HT_1B_R agonists, such as buspirone, improve depression/anxiety and sleeplessness [42,43,44]. TNF-α and IL-6, which are expressed through the activation of NF-κB [45], suppress the release of GABA from the hippocampus [46]. TNF-α does not suppress serotonin release directly, but it can influence serotonin level, and may play an important role in mood and inflammation [47,48]. Serotonin suppresses TNF-α expression [49,50]. These findings suggest that *B. longum* P77 can alleviate depression/anxiety and sleeplessness through the regulation of GABA and serotonin signaling, along with NF-κB activation.

*Bifidobacterium infantis* CCFM687, *B. longum* 1714, and *Bifidobacterium adolescentis* NK98 attenuate depression-like behavior in stress-exposed mice [10,16,29]. *L. reuteri* NK33, *L. plantarum* R6-3, and *L. plantarum* P72 alleviate depression-like symptoms in stress-exposed mice [26,29,51]. NVP1704, a combination of *L. reuteri* NK33 and *Bifidobacterium adolescentis* NK98, effectively alleviates depression- and insomnia-related symptoms in mice exposed to immobilization stress and healthy volunteers with depression and insomnia symptoms. In the present study, we found that *L. plantarum* P72 alleviated depression/anxiety- and sleeplessness-like behaviors, as previously reported [26]. *L. plantarum* P72 also increased immobilization stress- or cFM-suppressed GABA, serotonin, GABA_A_Rα1, GABA_A_Rα2, 5-HT_1A_R, and 5-HT_1B_R expression in the prefrontal cortex, like *B. longum* P77. Therefore, to understand whether the combination of *Lactobacillus* species and *Bifidobacterium* species could show the synergistic or antagonistic effects against depression and SN in vivo, we examined the combined effects of *L. plantarum* P72 and *B. longum* P77 on immobilization stress- or cFM-induced depression and SN in mice. When *B. longum* P77 (0.2 × 10^9^ CFU/mouse/day) was combined with *L. plantarum* P72 (0.8 × 10^9^ CFU/mouse/day), the combined P7277 (1 × 10^9^ CFU/mouse/day) alleviated additively or synergistically immobilization stress- or cFM-induced depression/anxiety- and sleeplessness-like behaviors in mice. P7277, a mix of *L. plantarum* P72 and *B. longum* P77, more potently, but not significantly, alleviated immobilization stress- or cFM-induced depression/anxiety- and sleeplessness-like behaviors than *B. longum* P77 (0.2 × 10^9^ CFU/mouse/day) or *L. plantarum* P72 (0.2 × 10^9^ CFU/mouse/day) alone. P7277 upregulated immobilization stress- or cFM-suppressed GABA, serotonin, GABA_A_Rα1, GABA_A_Rα2, 5-HT_1A_R, and 5-HT_1B_R expression in the brain more potently, but not significantly, than *L. plantarum* P72 or *B. longum* P77 alone. Moreover, P7277 also downregulated immobilization stress- orc cFM-induced corticosterone, TNF-α, IL-6, and NF-κB^+^ cell population in the brain and colon more potently, but not significantly, than *L. plantarum* P72 or *B. longum* P77 alone. These results suggest that the combination of *B. longum* P77 and *L. plantarum* P72 may additively or synergistically alleviate depression and sleeplessness and could therefore offer greater therapeutic benefits for the treatment of depression, anxiety, and sleeplessness than either probiotic alone.

hP7277, a heat-killed P7277, also alleviated depression/anxiety- and sleeplessness-like behaviors. hP7277 also upregulated immobilization stress-suppressed GABA, serotonin, GABA_A_Rα1, GABA_A_Rα2, 5-HT_1A_R, and 5-HT_1B_R expression in the brain and downregulated immobilization stress-induced corticosterone, TNF-α and IL-6 levels in the brain and colon. However, its efficacy was weaker than that of live P7277. These results suggest that, although the reduced efficacy of hP7277 may be because it lacks the ability to proliferate or adhere in the intestine, it can also be useful as an alternative therapeutic option of depression/anxiety and sleeplessness in place of live P7277.

Approximately 90% of patients with depression suffer from insomnia [6,52]. Nevertheless, insomnia can sometimes be an early warning sign that precedes the development of depression. Therefore, combing standard antidepressant medication with sleep-focused interventions has shown significant success in improving both depression and insomnia [53]. Based on these findings, P7277 may be beneficial for the therapy of patients with depression and sleeplessness (insomnia).

In the present study, we were not able to investigate whether *B. longum* P77, *L. plantrum* P72, and P7277 are effective as a single-dose treatment or whether their effects differ by sex. Therefore, further studies including these aspects are necessary to clarify the specific mechanisms through which the components of *L. plantarum* P72 and *B. longum* P77 exert their effects on depression and sleeplessness.

## 5. Conclusions

*B. longum* P77 alleviated depression/anxiety- and sleeplessness-like symptoms by inducing GABA and serotonin production and suppressing the expression of proinflammatory cytokines. P7277, a mix of *L. plantarum* P72 and *B. longum* P77, additively or synergistically alleviated depression/anxiety- and sleeplessness-like symptoms. P7277 and hP7277, which is a heat-killed P7277, may improve depression/anxiety and sleeplessness through the activation of GABA and serotonin systems, along with the suppression of NF-κB activation.

## Data Availability

The data generated in this study are available upon request from the corresponding author.

## Supplementary Materials

The following supporting information can be downloaded at: https://www.mdpi.com/article/10.3390/cells14191547/s1, Figure S1. Taxonomic classification by genome-wide comparative analysis of *B. longum* P77; Figure S2. Effects of *B. longum* P77 and diphenhydramine on GABA_A_Rα1^+^ (a) and NF-κB^+^CD11c^+^ cell population (b) in the prefrontal cortex of mice with immobilization stress-induced depression/anxiety and sleeplessness; Figure S3. Effects of *B. longum* P77 and diphenhyramine on colonic NF-κB^+^CD11c^+^ cell population in mice with immobilization stress-induced colitis; Table S1: Primers used in this study; Table S2. Statistic data in the present study. Methods: Preparation of mice with immobilization stress and behavioral tasks.
