# Supplementary material for: Bifidobacterium longum P77 and Lactiplantibacillus plantarum P72 and Their Mix—Live or Heat-Treated—Mitigate Sleeplessness and Depression in Mice: Involvement of Serotonergic and GABAergic Systems
Source: Cells. 2025 Oct 3;14(19):1547. doi: 10.3390/cells14191547 (PMC12523862; doi:10.3390/cells14191547)
Supplement: Supplementary file 1 [file cells-14-01547-s001.zip › cells-3825827-supplementary.pdf]

Bifidobacterium longum P77 and _Lactiplantibacillus plantarum_ P72, and their mix – live or heat-treated - mitigate sleeplessness and depression in mice: Involvement of serotonergic and GABAergic systems

Table S1. Primers used in this study

gene	Primer	
	Forward	Reverse
$GABA_A$ receptor α1	5′- GAGTCGTCCAATCCAGCAC-3′	5′- AGCCAGAAGGAAACCTGTGA-3′
$GABA_A$ receptor α2	5′- TTACAGTCCAAGCCGAATGTCCC-3′	5′- ACTTCTGAGGTTGTGTAAGCGTAGC-3′
5-HT_{1A}R	5′- CCGTGAGAGGAAGACAGTCTAAGA-3′	5′- GGTTGAGCAGGGAGTTGGAGTAG-3′
5-HT_{1B}R	5′- CCAGCGGTCCATCCACAGAG-3′	5′- CCAGCGGTCCATCCACAGAG-3′
GAPDH	5′-TGCAGTGGCAAAGTGGAGAT-3′	5′-TTTGCCGTGAGTGGAGTCATA-3′

Table S2. Statistic data in the present study

Figure 1. One-way ANOVA with Tukey multiple comparisons test.			
	NC vs. Cort	NC vs. P77	Cort vs. P77
(a) Serotonin(pg/mL) P<0.0001, η^2= 0.840	P<0.0001	P=0.0002	P<0.0001
Cohen's d	3.73	-2.18	-4.91

Figure 2. One-way ANOVA with Tukey multiple comparisons test.						
	NC vs. IM	NC vs. P77	NC vs. DH	IM vs. P77	IM vs. DH	P77 vs. DH
(a) Total distance moved(m) P<0.0001, η^2=0.784	P<0.0001	P=0.4629	P<0.0001	P<0.0001	P=0.9998	P<0.0001
Cohen's d	3.22	-0.62	3.75	-3	0.06	3.82

				.42		
(b) Distance traveled in the center(m) P <0.0001, η^2=0.756	P=0.0010	P=0.0045	P=0.0021	P<0.0001	P=0.9932	P<0.0001
Cohen’s d	1.71	-1.49	1.75	-4.90	-0.20	5.97
(c) Time spent in the center(s) P<0.0001, η^2=0.770	P<0.0001	P=0.9935	P<0.0001	P<0.0001	P=0.9168	P<0.0001
Cohen’s d	2.81	0.10	2.52	-6.70	-1.00	5.58
(e) Time spent in the open arms(%) P<0.0001, η^2=0.845	P<0.0001	P=0.7607	P<0.0001	P<0.0001	P=0.9632	P<0.0001
Cohen’s d	3.60	0.42	4.13	-4.80	0.30	6.32
(f) Entry number into the open arms(%) P<0.0001, η^2=0.832	P<0.0001	P<0.0001	P<0.0001	P=0.00	P=0.8524	P=0.0010

				80		
Cohen’s d	4.74	2.86	5.85	-1.61	0.45	2.22
(g) Immobility times(s) P<0.0001, η^2=0.827	P<0.0001	P=0.5284	P<0.0001	P<0.0001	P=0.9998	P<0.0001
Cohen’s d	-4.93	-1.14	-4.23	3.90	0.03	-3.39
(h) Sleep latency time(s) P<0.0001, η^2=0.808	P<0.0001	P=0.0065	P<0.0001	P<0.0001	P=0.2254	P=0.0009
Cohen’s d	-4.66	-1.87	-4.12	3.04	0.95	-2.32
(i) Sleep duration(m) P<0.0001, η^2=0.871	P<0.0001	P=0.0001	P=0.0570	P=0.0002	P<0.0001	P<0.0001
Cohen’s d	4.23	1.91	-1.07	-3.71	-12.60	-5.02

Figure 3. One-way ANOVA with Tukey multiple comparisons test.

	NC vs. IM	NC vs. P77	NC vs. DH	IM vs. P77	IM vs. DH	P77 vs. DH

(a) GABA(pg/mg)<br>P<0.0001, η^2=0.724	P<0.0001	P=0.0539	P=0.0355	P=0.0015	P=0.0023	P=0.9970
Cohen's d	3.40	1.32	1.58	-2.79	-3.33	0.16
(b) $GABA_AR\alpha1$(fold change)<br>P=0.0002, η^2=0.611	P=0.0012	P=0.6917	P=0.0014	P=0.0149	P>0.9999	P=0.0170
Cohen's d	2.10	0.48	2.14	-2.54	-0.07	2.73
(c) $GABA_AR\alpha2$(fold change)<br>P<0.0001, η^2=0.629	P<0.0001	P=0.1575	P=0.0162	P=0.0109	P=0.1139	P=0.6752
Cohen's d	3.47	1.77	1.95	-2.00	-1.13	0.62
(e) Serotonin(pg/mg)<br>P=0.0001, η^2=0.635	P=0.0025	P=0.5970	P=0.1416	P=0.0001	P=0.2569	P=0.0106
Cohen's d	3.43	-0.89	1.31	-3.11	-0.95	1.65
(f) $5\text{-}HT_{1A}R$<br>P<0.0001, η^2=0.747	P=0.0007	P=0.3714	P=0.0024	P<0.0001	P=0.9408	P<0.0001
Cohen's d	2.53	-0.74	2.20	-4.11	-0.59	3.70
(g) $5\text{-}HT_{1B}R$<br>P<0.0001, η^2=0.752	P<0.0001	P=0.0066	P<0.0001	P=0.0631	P=0.9488	P=0.0205
Cohen's d	4.17	1.94	4.11	-1.51	0.35	1.69
(h) Corticosterone(ng/mg)<br>P<0.0001, η^2=0.772	P=0.0011	P=0.1018	P=0.0026	p<0.0001	P=0.9809	P<0.0001
Cohen's d	-3.08	1.40	-2.12	4.71	0.22	-3.36
(i) TNF-α(ng/mg)<br>P<0.0001, η^2=0.560	P=0.0125	P>0.9999	P>0.9999	P=0.0006	P=0.1672	P=0.1323
Cohen's d	-2.10	0.79	-0.92	2.31	1.33	-1.40
(j)IL-6(pg/mg)<br>P<0.0001, η^2=0.517	P=0.0014	P=0.4980	P=0.0454	P=0.0345	P=0.4230	P=0.5027
Cohen's d	-3.59	-0.98	-1.69	1.67	0.79	-0.67
(k)IL-10(ng/mg)<br>P<0.0001, η^2=0.772	P<0.0001	P=0.0309	P<0.0001	P=0.0019	P=0.8177	P=0.0134
Cohen's d	4.59	1.42	3.44	-2.72	-0.73	1.84

Figure 4. One-way ANOVA with Tukey multiple comparisons test.

	NC vs. IM	NC vs. P77	NC vs. DH	IM vs. P77	IM vs. DH	P77 vs. DH
(a) Myeloperoxidase(pg/mg) P=0.0001, η^2= 0.586	P=0.0016	P=0.5430	P=0.0020	P=0.0316	P=0.9995	P=0.0396
Cohen's d	-3.51	-0.90	-2.34	1.85	0.05	-1.38
(b) TNF-α(ng/mg) P=0.0001, η^2= 0.628	P=0.0003	P=0.3576	P=0.0013	P=0.0144	P=0.9256	P=0.0533
Cohen's d	-5.26	-1.03	-2.34	2.19	0.34	-1.23
(c) IL-1β (pg/mg) P=0.0013, η^2=0.535	P=0.0007	P=0.1247	P=0.0279	P=0.1116	P=0.3799	P=0.8761
Cohen's d	-2.77	-2.28	-1.75	1.41	0.74	-0.43
(d) IL-6(pg/mg) P<0.0001, η^2=0.679	P=0.0002	P=0.9789	P=0.0348	P<0.0001	P=0.1370	P=0.0149
Cohen's d	-2.73	0.41	-1.85	3.05	1.01	-2.22
(e) IL-10(ng/mg) P=0.715, η^2=12.55	P=0.0001	P=0.0349	P<0.0001	P=0.0737	P=0.8378	P=0.0127
Cohen's d	3.76	1.64	2.98	-2.21	0.51	1.76

Figure 5. One-way ANOVA with Tukey multiple comparisons test.

	NC vs FM	NC vs. P72	NC vs. P77	NC vs P72P77	FM vs. P72	FM vs P77	FM vs. P72P77	P72 vs. P77	P72 vs. P72P77	P77 vs. P72P77
(a) Total distance moved(m) P=0.0006, η^2=0.423	P=0.0003	P=0.8988	P=0.1296	P=0.3572	P=0.0044	P=0.1743	P=0.0518	P=0.5407	P=0.8667	P=0.9777
Cohen's d	4.93	0.33	2.00	1.29	-1.49	-2.32	-1.98	0.58	-0.20	-0.35
(b) Distance traveled in the center(m) P<0.0001, η^2= 0.651	P<0.0001	P=0.7294	P=0.9785	P=0.6584	P<0.0001	P<0.0001	P=0.0010	P=0.9620	P >0.9999	P=0.9329
Cohen's d	4.42	0.61	0.38	0.59	-3.22	-5.61	-2.46	-0.39	0.30	0.35
(c) Time spent in th center(s)	P<0.0001	P= 0.0005	P=0.0007	P=0.0027	P<0.0001	P<0.0001	P<0.0001	P>0.9999	P=0.9757	P=0.9757

P<0.0001, η^2= 0.765										
Cohen’s d	3.91	1.83	1.83	2.34	-3.33	-3.55	-3.84	-0.08	-0.35	-0.38
(e) Time spent in the open arms(%)<br>P=0.0002, η^2=0.461	P=0.0022	P=0.9909	P=0.9993	P>0.9999	P=0.0002	P=0.0142	P=0.0032	P=0.7471	P=0.9762	P=0.9999
Cohen’s d	1.75	-0.37	0.29	0.08	-2.50	-1.94	-2.42	0.83	-0.30	-0.35
(f) Entry number into the open arms(%)<br>P<0.0001, η^2=0.672	P<0.0001	P= 0.8342	P=0.7443	P=0.9984	P<0.0001	P<0.0001	P<0.0001	P=0.1842	P=0.9427	P=0.5673
Cohen’s d	2.99	-0.68	0.90	-0.11	-3.77	-2.84	-2.47	2.15	-0.66	-0.70
(g) Immobility times(s)<br>P<0.0001, η^2=0.600	P=0.0063	P=0.0321	P=0.7939	P=0.4286	P<0.0001	P=0.0003	P<0.0001	P=0.3162	P=0.6746	P=0.9727
Cohen’s d	-2.69	1.95	0.60	0.82	3.58	2.26	2.57	-0.85	0.27	0.25
(h) Sleep latency time(s)<br>P<0.0001, η^2=0.583	P=0.0002	P= 0.6586	P=0.3831	P=0.4348	P=0.0080	P=0.0260	P<0.0001	P=0.9907	P=0.0309	P=0.0097
Cohen’s d	-2.54	-0.86	-0.86	0.90	1.59	1.18	3.48	-0.19	2.31	1.71
(i) Sleep duration(m)<br>P<0.0001, η^2=0.515	P<0.0001	P=0.0884	P=0.0578	P=0.9065	P=0.9065	P=0.0378	P=0.0590	P=0.9997	P=0.4170	P=0.3144
Cohen’s d	6.94	1.62	1.98	0.36	-1.91	-2.05	-1.97	0.10	-0.70	-0.73

Figure 6. One-way ANOVA with Tukey multiple comparisons test.

	NC vs. FM	NC vs. P72	NC vs. P77	NC vs. P72P77	FM vs. P72	FM vs. P77	FM vs. P72P77	P72 vs. P77	P72 vs. P72P77	P77 vs. P72P77
(a) GABA(pg/mg)<br>P<0.0001, η^2=0.666	P<0.0001	P=0.9959	P=0.9487	P>0.9999	P=0.0001	P=0.0003	P<0.0001	P=0.9962	P=0.9946	P=0.9414
Cohen’s d	2.75	0.17	0.37	-0.02	-3.22	-3.40	-3.98	0.24	-0.51	-0.60
(b $GABA_A R\alpha1$(fold change)	P<0.0001	P=0.9120	P=0.9919	P=0.4057	P=0.0004	P=0.0001	P<0.0001	P=0.9935	P=0.0946	P=0.2055

P<0.0001, η²=0.719										
Cohen's d	2.91	0.68	0.32	-0.80	-2.52	-2.63	-3.42	-0.31	-1.40	-1.32
(c) $GABA_AR\alpha2$(fold change)<br>P<0.0001, η²=0.654	P=0.0007	P>0.9999	P=0.9982	P=0.4938	P=0.0005	P=0.0003	P<0.0001	P=0.9996	P=0.5540	P=0.6749
Cohen's d	3.12	-0.06	-0.19	-0.90	-2.82	-3.00	-3.51	-0.11	-0.70	-0.71
(e) Serotonin(pg/mg)<br>P=0.0063, η²=0.424	P=0.0321	P= 0.9875	P=0.9941	P=0.9852	P=0.0102	P=0.0766	P=0.0097	P=0.8970	P>0.9999	P=0.8882
Cohen's d	2.21	-0.28	0.23	-0.30	-2.56	-1.97	-2.12	0.52	-0.46	-0.46
(f) $5\text{-}HT_{1A}R$<br>P=0.0035, η²=0.453	P= 0.0034	P= 0.9968	P= 0.4039	P= 0.6385	P= 0.0078	P= 0.1786	P= 0.0837	P= 0.6080	P= 0.8308	P= 0.9946
Cohen's d	2.75	0.24	1.61	0.64	-2.05	-1.44	-1.24	0.94	-0.19	-0.20
(g) $5\text{-}HT_{1B}R$<br>P<0.0001, η²=0.631	P= 0.0007	P= 0.7340	P= 0.3443	P>0.9999	P <0.0001	P= 0.0690	P= 0.0006	P= 0.0328	P= 0.7681	P= 0.3143
Cohen's d	2.54	-0.74	1.05	-0.03	-4.48	-1.85	-2.46	2.49	-1.12	-1.04
(h) Corticosterone(ng/mg)<br>P<0.0001, η²= 0.740	P<0.0001	P= 0.0519	P= 0.5718	P= 0.2905	P= 0.0004	P<0.0001	P<0.0001	P= 0.6215	P= 0.8905	P= 0.9856
Cohen's d	-4.70	-2.63	-0.86	-1.00	3.22	3.11	2.90	0.88	-0.32	-0.25
(i) TNF-α(pg/mg)<br>P=0.0009, η²= 0.512	P= 0.0011	P= 0.2092	P= 0.4605	P= 0.9986	P= 0.1728	P= 0.0614	P=0.0022	P=0.9841	P=0.3287	P=0.6300
Cohen's d	-3.14	-1.39	-1.10	-0.14	1.84	2.38	2.09	0.38	0.66	0.67
(j) IL-10(ng/mg)<br>P<0.0001, η²= 0.622	P <0.0001	P=0.4848	P=0.0645	P=0.5193	P=0.0014	P=0.0249	P=0.0012	P=0.7660	P >0.9999	P=0.7338
Cohen's d	3.04	0.70	1.65	1.23	-2.14	-2.58	-3.48	0.66	-0.68	-1.60
	P<0.0001	P=0.0884	P=0.0578	P=0.9065	P=0.9065	P=0.0378	P=0.0590	P=0.9997	P=0.4170	P=0.3144

Figure 7. One-way ANOVA with Tukey multiple comparisons test.

	NC vs. FM	NC vs. P72	NC vs. P77	NC vs. P72P77	FM vs. P72	FM vs. P77	FM vs. P72P77	P72 vs. P77	P72 vs. P72P77	P77 vs. P72P77
(a) Myeloperoxidase(pg/mg) P=0.0014, η^2=0.494	P=0.0336	P=0.5879	P=0.9980	P=0.9441	P=0.0009	P=0.0650	P=0.0058	P=0.4086	P=0.9472	P=0.8317
Cohen's d	-2.40	1.08	-0.19	0.41	2.63	1.46	2.11	-0.90	0.56	0.51
(b) TNF-α(pg/mg) P=0.0001, η^2=0.593	P=0.0013	P>0.9999	P>0.9999	P=0.9216	P=0.0015	P=0.0010	P=0.0002	P=0.9998	P=0.9032	P=0.9511
Cohen's d	-2.26	0.39	-0.63	0.46	2.83	1.81	2.43	-1.20	1.18	1.07
(c) IL-1β (pg/mg) P=0.0001, η^2=0.591	P=0.0007	P>0.9999	P=0.9982	P=0.4938	P=0.0005	P=0.0003	P<0.0001	P=0.9996	P=0.5540	P=0.6749
Cohen's d	-2.75	-0.04	0.07	0.41	2.37	2.21	2.65	0.09	0.42	0.37
(d) IL-6(ng/mg) P=0.0016, η^2=0.489	P=0.0053	P>0.9999	P=0.9993	P>0.9999	P=0.0062	P=0.0092	P=0.0038	P=0.9998	P>0.9996	P=0.9963
Cohen's d	-1.70	-0.05	-0.17	0.06	1.75	1.63	1.79	-0.13	0.30	0.28
(e) IL-10(ng/mg) P<0.0001, η^2=0.390	P=0.0332	P=0.9863	P=0.9944	P=0.9937	P=0.0103	P=0.0782	P=0.0802	P=0.8940	P=0.8895	P>0.9999
Cohen's d	2.08	-0.26	0.31	0.25	-1.64	-1.68	-1.67	0.44	0.01	0.01

Figure 8. One-way ANOVA with Tukey multiple comparisons test.

	NC vs. IM	NC vs. P72	NC vs. P77	NC vs. P72P77	IM vs. P72	IM vs. P77	IM vs. P72P77	P72 vs. P77	P72 vs. P72P77	P77 vs. P72P77
(a) Total distance moved(m) P<0.0001, η^2=0.642	P=0.0018	P=0.1348	P=0.7595	P=0.0545	P<0.0001	P<0.0001	P<0.0001	P=0.7338	P=0.9931	P=0.4754
Cohen's d	2.59	-0.86	-0.74	-2.08	-2.72	-7.08	-5.12	0.51	-0.62	-1.21
(b) Distance traveled in the center(m) P=0.0001, η^2=0.468	P=0.0014	P>0.9999	P>0.9999	P=0.9765	P=0.0020	P=0.0015	P=0.0002	P>>0.9999	P=0.9487	P=0.9703

Cohen's d	1.95	0.07	0.02	-0.31	-3.58	-2.66	-2.55	-0.07	-0.33	-0.29
(c) Time spent in th center(s)<br>P<0.0001, η^2=0.615	P<0.0001	P=0.9502	P=0.9752	P=0.8157	P<0.0001	P<0.0001	P<0.0001	P=0.6844	P=0.9963	P=0.4633
Cohen's d	1.55	-1.10	-0.43	-1.55	-4.92	-3.56	-4.09	1.03	-0.65	-0.64
(e) Time spent in the open arms(%)<br>P<0.0001, η^2=0.706	P=0.0022	P=0.9909	P=0.9993	P>0.9999	P=0.0002	P=0.0142	P=0.0032	P=0.7471	P=0.9762	P=0.9999
Cohen's d	3.75	-0.34	0.22	-1.07	-4.85	-2.77	-7.83	0.52	-1.12	-0.76
(f) Entry number into the open arms(%)<br>P<0.0001, η^2=0.676	P<0.0001	P=0.0587	P=0.0150	P=0.6462	P<0.0001	P=0.0005	p<0.0001	P=0.9798	P=0.6142	P=0.2923
Cohen's d	3.28	1.73	2.09	0.52	-2.55	-2.29	-2.50	0.64	-1.03	-1.04
(g) Immobility times(s)<br>P<0.0001, η^2=0.765	P<0.0001	P=0.6643	P=0.0071	P=0.7159	P<0.0001	P<0.0001	P<0.0001	P=0.1681	P>0.9999	P=0.1424
Cohen's d	-5.38	-0.69	-2.37	-0.53	4.63	4.50	3.43	-1.50	0.94	1.07
(h) Sleep latency time(s)<br>P<0.0001, η^2=0.524	P=0.0003	P=0.9885	P=0.9966	P=0.9987	P<0.0001	P=0.0008	P=0.0001	P=0.9183	P=0.9996	P=0.9700
Cohen's d	-2.70	-1.21	-0.55	0.56	1.92	2.03	3.94	0.53	1.54	1.15
(i) Sleep duration(m)<br>P=0.0107, η^2=0.305	P=0.001	P=0.3099	P=0.5034	P=0.0556	P=0.0089	P=0.0211	P=0.0007	P=0.7253	P=0.3487	P=0.2007
Cohen's d	1.08	-0.37	0.56	-1.21	-1.10	-0.65	-2.29	0.77	-1.03	-1.69

Figure 9. One-way ANOVA with Tukey multiple comparisons test.

	NC vs. IM	NC vs. P72	NC vs. P77	NC vs. P72P77	IM vs. P72	IM vs. P77	IM vs. P72P77	P72 vs. P77	P72 vs. P72P77	P77 vs. P72P77
(a) GABA(pg/mg)<br>P<0.0001, η^2=0.605	P=0.0004	P=0.9941	P=0.9487	P=0.9689	P=0.0012	P=0.0027	P<0.0001	P=0.9976	P=0.8383	P=0.6629
Cohen's d	3.38	0.19	0.40	-0.55	-2.35	-2.63	-4.85	0.15	-0.71	-0.87
(b) $GABA_AR\alpha1$(fol	P=0.0	P=0.997	P=0.846	P=0.9	P=0.014	P=0.0	P=0.001	P=0.953	P=0.841	P=0.434

d change)<br>$P=0.0015$, $\eta^2=0.493$	068	9	4	509	4	698	1	6	2	8
Cohen’s d	2.57	0.19	0.54	-0.41	-2.85	-1.83	-2.60	0.42	-0.92	-0.83
(c) $GABA_AR\alpha2$(fold change)<br>$P=0.0005$, $\eta^2=0.536$	P=0.0002	P=0.1916	P=0.1606	P=0.0360	P=0.0394	P=0.0489	P=0.2056	P>0.9999	P=0.9198	P=0.9478
Cohen’s d	3.25	0.96	1.22	3.82	-1.66	-2.35	-2.75	0.05	0.39	0.56
(d) Serotonin(pg/mg)<br>$P<0.0001$, $\eta^2=0.693$	P=0.0008	P=0.7539	P=0.7688	P=0.1874	P<0.0001	P=0.0151	P<0.0001	P=0.1587	P=0.8167	P=0.0160
Cohen’s d	3.65	-0.62	0.88	-1.36	-3.01	-2.59	-4.14	1.19	-1.55	-2.00
(e) $5\text{-}HT_{1A}R$<br>$P<0.0001$, $\eta^2=0.462$	P=0.0103	P=0.9949	P=0.9074	P=0.9722	P=0.0256	P=0.0728	P=0.0023	P=0.9888	P=0.8542	P=0.5883
Cohen’s d	1.71	0.17	0.42	-0.40	-2.71	-3.47	-2.78	0.44	-0.87	-1.03
(f) $5\text{-}HT_{1B}R$<br>$P=0.0029$, $\eta^2=0.682$	P=0.0015	P=0.9777	P=0.9759	P=0.0584	P =0.0061	P=0.0063	P<0.0001	P>0.9999	P=0.0160	P=0.0156
Cohen’s d	2.27	0.31	0.26	-2.03	-3.20	-2.17	-5.15	0.01	-2.77	-1.92
(g) Corticosterone(ng/mg)<br>$P<0.0001$, $\eta^2=0.631$	P=0.0005	P=0.9076	P=0.9406	P=0.9561	P=0.0046	P<0.0001	P<0.0001	P=0.4995	P=0.5376	P>0.9999
Cohen’s d	-3.62	-0.53	0.34	0.63	3.18	2.81	5.02	0.74	-0.04	-0.03
(h) TNF-α(pg/mg)<br>$P=0.0220$, $\eta^2=0.356$	P=0.0176	P= 0.5798	P=0.3942	P=0.3024	P=0.0587	P=0.1065	P=0.0014	P=0.7621	P=0.1191	P=0.0663
Cohen’s d	-2.78	-0.72	-0.88	0.43	1.88	1.39	1.46	-0.28	0.79	0.77
(i) IL-10(ng/mg)<br>$P=0.0009$, $\eta^2=0.512$	P=0.0047	P=0.9481	P>0.9999	P=0.0425	P=0.0264	P=0.0056	P=0.8789	P=0.9644	P=0.1834	P=0.0499
Cohen’s d	2.68	0.36	0.05	2.08	-1.44	-2.24	-0.65	-0.30	1.48	2.13

Figure 10. One-way ANOVA with Tukey multiple comparisons test.

	NC vs. IM	NC vs. P72	NC vs. P77	NC vs. P72P77	IM vs. P72	IM vs. P77	IM vs. P72P77	P72 vs. P77	P72 vs. P72P77	P77 vs. P72P77
(a) Myeloperoxidas	P=0.0	P=0.286	P=0.892	P=0.5	P=0.000	P=0.0	P=0.000	P=0.797	P=0.984	P=0.975

e(ng/mg)<br>P=0.0002, η^2=0.569	337	5	4	739	2	039	9	7	3	0
Cohen’s d	-1.60	3.20	1.07	0.59	2.54	1.91	1.82	-1.12	0.34	0.32
(b) TNF-α(ng/mg)<br>P=0.0057, η^2=0.429	P=0.0091	P=0.2063	P=0.9760	P=0.8266	P=0.0004	P=0.0085	P=0.0054	P=0.2168	P=0.2921	P=0.8501
Cohen’s d	-2.08	1.10	0.03	0.10	2.65	1.86	1.34	-0.91	0.09	0.09
(c) IL-1β (ng/mg)<br>P=0.0003, η^2=0.559	P=0.0013	P=0.9973	P=0.9976	P=0.9998	P=0.0006	P=0.0029	P=0.0020	P=0.9655	P=0.9867	P=0.9999
Cohen’s d	-2.34	0.33	-0.32	-0.07	2.25	1.95	1.82	-0.47	0.09	0.09
(d) IL-6(pg/mg)<br>P<0.0001, η^2=0.675	P=0.0118	P=0.0919	P=0.3417	P=0.2219	P<0.0001	P=0.0001	P<0.0001	P=0.9441	P=0.9893	P=0.9987
Cohen’s d	-1.58	1.61	1.24	1.08	2.89	2.64	2.86	-0.54	0.21	0.24
(e) IL-10(ng/mg)<br>P<0.0001, η^2=0.620	P<0.0001	P=0.5936	P=0.1548	P=0.0017	P=0.0025	P=0.0228	P=0.6915	P=0.8894	P=0.0545	P=0.3023
Cohen’s d	4.10	0.90	1.71	2.05	-3.61	-4.06	-0.61	0.78	0.87	0.93

Figure11. One-way ANOVA with Tukey multiple comparisons test.

	NC vs. IM	NC vs. hP72P77	IM vs. hP72P77
(a) Total distance moved(m)<br>P=0.0208, η^2=0.308	P=0.0240	P=0.8683	P=0.0691
Cohen’s d	2.94	0.30	-2.36
(b) Distance traveled<br>in the center(m)<br>P=0.0088, η^2=0.363	P=0.0023	P=0.0840	P=0.0148
Cohen’s d	3.27	1.68	-1.30
(c) Time spent in the center(s)<br>P=0.0056, η^2=0.640	P=0.0040	P=0.2520	P=0.0321
Cohen’s d	-3.39	-0.12	3.20
(e) Time spent in the open arms(%)<br>P<0.0001, η^2=0.640	P<0.0001	P=0.8247	P=0.0002
Cohen’s d	1.44	0.25	-1.17

(f) Entry number into the open arms(%)<br>P<0.0001, η^2=0.648	P<0.0001	P=0.0011	P=0.0268
Cohen’s d	1.54	0.84	-1.10
(g) Immobility times(s)<br>P<0.0001, η^2=0.744	P=0.0337	P=0.9706	P<0.0001
Cohen’s d	1.62	0.73	-1.50
(h) Sleep latency time(s)<br>P=0.0023, η^2=0.438	P=0.0031	P=0.8213	P=0.0124
Cohen’s d	-1.82	-0.33	1.51
(i) Sleep duration(m)<br>P=0.0032, η^2=0.420	P=0.0089	P=0.9900	P=0.0065
Cohen’s d	1.64	-0.06	-1.94
Figure12. One-way ANOVA with Tukey multiple comparisons test.			
	NC vs. hP72P77	IM vs. hP72P77	NC vs. hP72P77
(a) Serotonin(pg/mg)<br>P<0.0001, η^2=0.724	P<0.0001	P=0.0695	P=0.0010
Cohen’s d	3.33	1.21	-2.80
(b) $5\text{-}HT_{1A}R$<br>P=0.0010, η^2=0.534	P=0.0243	P=0.2829	P=0.0008
Cohen’s d	1.58	-0.73	-2.81
(c) Corticosterone(ng/mg)<br>P=0.0007, η^2=0.555	P=0.0005	P=0.0501	P=0.1003
Cohen’s d	-2.50	-1.25	1.33
(d) TNF-α(pg/mg)<br>P=0.0044, η^2=0.452	P=0.0070	P=0.9438	P=0.0140
Cohen’s d	-1.83	-0.21	1.51
(e) IL-6(pg/mg)<br>P=0.0034, η^2=0.467	P=0.0062	P=0.9723	P=0.0100
Cohen’s d	-1.79	-0.15	1.60
(f) IL-10(pg/mg)	P=0.0332	P=0.9910	P=0.0255

P=0.0154, η^2=0.370			
Cohen’s d	1.39	-0.06	-1.84

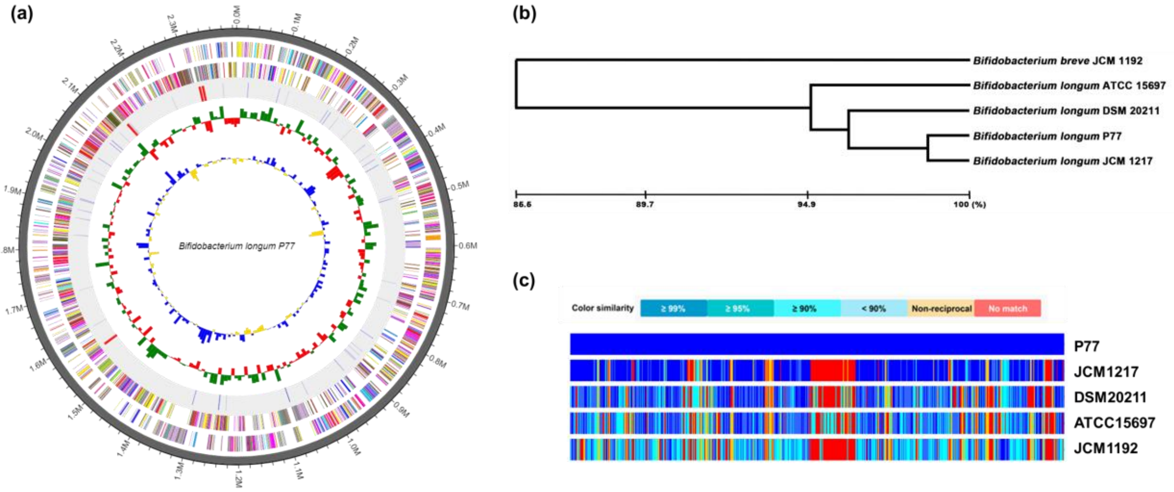

Figure S1. Taxonomic classification by genome-wide comparative analysis of *B. longum* P77. (a) Neighbor-joining tree based on the OrthoANI distance matrix (analyzed using UPGMA dendrogram, Newick format). (b) The pairwise ortholog matrix table (generated and colored according to the similarity between matching sequences).

(a)

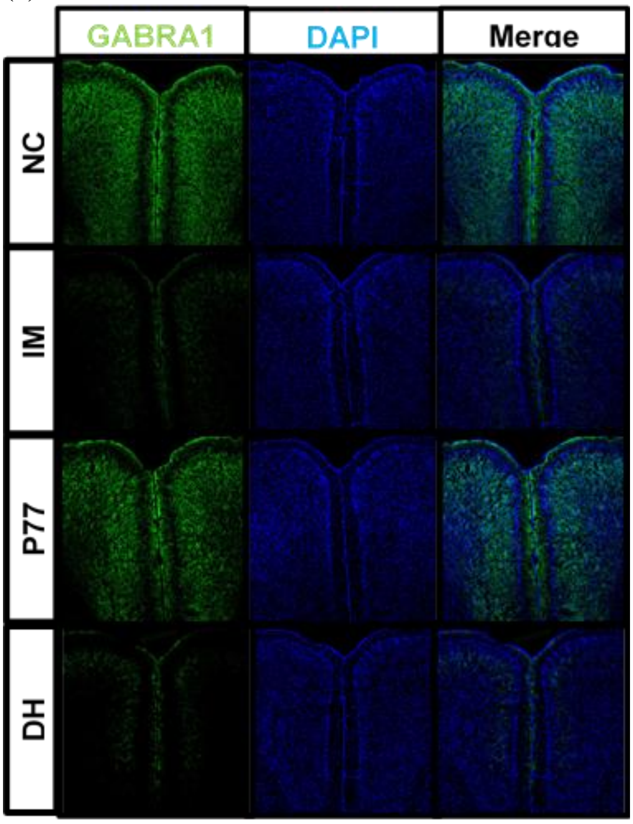

(b)

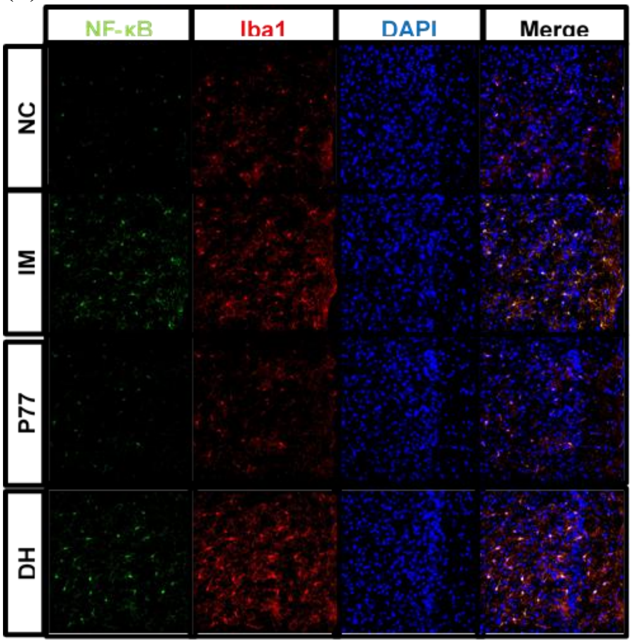

Figure S2. Effects of *B. longum* P77 and diphenhydramine on $GABA_AR\alpha1^+$ (a) and NF-κB^+CD11c^+ cell population (b) in the prefrontal cortex of mice with immobilization stress-induced depression/anxiety and sleeplessness. Test agents (IM, vehicle; P77, 1×10^9 CFU/mouse/day of *B. longum* P77; DH, 20 mg/kg of diphenhydramine) were treated in immobilization stress/pentobarbital-exposed mice. NC was treated with saline in immobilization stress-untreated mice. n=8. $^{\#}p<0.05$ vs NC. $^{*}p<0.05$ vs IM.

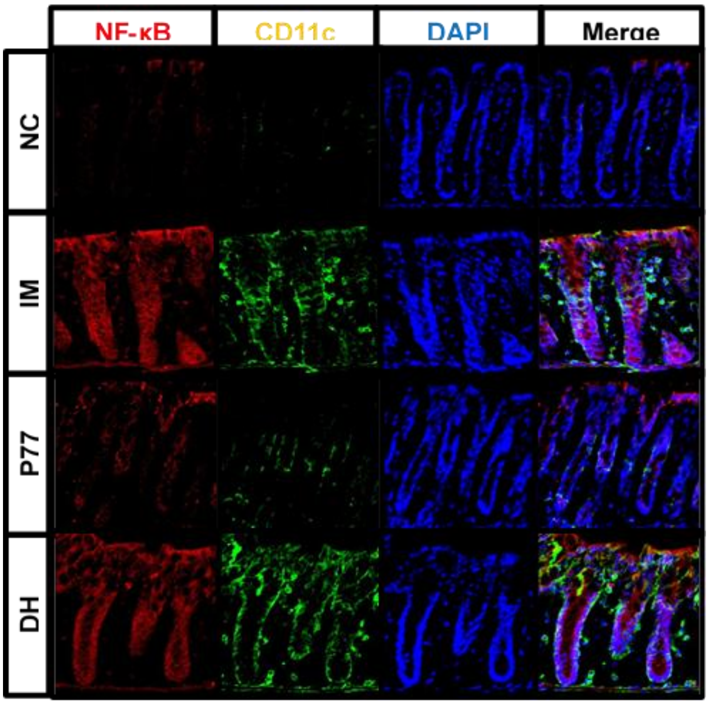

Figure S3. Effects of *B. longum* P77 and diphenhydramine on colonic NF-κB$^+$CD11c$^+$ cell population in mice with immobilization stress-induced colitis. Test agents (IM, vehicle; P77, 1x10^9 CFU/mouse/day of *B. longum* P77; DH, 20 mg/kg of diphenhydramine) were treated in immobilization stress/pentobarbital-exposed mice. NC was treated with saline in immobilization stress-untreated mice. n=8. $^{\#}p<0.05$ vs NC. $^{*}p<0.05$ vs IM.

Methods

Preparation of mice with immobilization stress

Immobilization stress was performed, as follows: each mouse was inserted into a conical tube-like instrument (2.5 cm in diameter, 7.5 cm in length) with a 0.25-cm-diameter hole on the center of the tube) and vertically placed for 8 h/day and repeated for 5 days, as previously reported (Jang et al., 2018).

Behavioral tasks

Depression- and sleepless-like behaviors were measured in a room equipped with a record camera and quantified using the EthoVision XT software. For the assay of depression-like behaviors, the open field test was performed in a chamber (40×40 cm; center zone, 20×20 cm) for 10 min (Joo et al., 2023). The elevated plus maze test was measured in a plus maze apparatus consisting of two open [30 × 7 cm] and two enclosed [30 × 7 cm] arm with 20-cm-high walls extending from a central platform [7 × 7 cm] (Jang et al., 2019). The TST was measured on the edge of a table, at 30 cm above it (Jang et al., 2019).

Sleeping tests for sleep latency time and sleep duration were measured under the exposure to pentobarbital sodium or isoflurane (Ma et al., 2009). Pentobarbital sodium (40 mg/kg, Hanlim Pharm. Co., Ltd., Korea, diluted in sterilized saline) was intraperitoneally injected or isofurane (2%) in a chamber was exposed for 10 min from next day after the final depression-like behavioral task. Diphenhydramine (20 mg/kg) was orally gavaged 30 min before the

injection of pentobarbital sodium. The time of righting reflex loss and its appearance were measured for 10 and 60 min in isofurane- and pentobarbital-induced sleep test, respectively. Latency time for righting reflex sleep latency time and sleep duration (the time from righting reflex loss to righting reflex recovery) were measured.

Enzyme-linked immunosorbent assay

Collected brain (prefrontal cortex) and colon tissues were homogenized and lysed in RIPA buffer (150 mM sodium chloride, 1% sodium deoxycholate, 1% Triton X-100, 0.1% SDS, 50 mM Tris–HCl, 2 mM EDTA, pH 7.5) containing a phosphatase inhibitor cocktail (Roche) and centrifuged at 10,000 g and 4°C for 20 min, as previously reported (Joo et al., 2023). In the supernatant, TNF-α, IL-1β, IL-6, IL-10, and myeloperoxidase (R&D system, Minneapolis, Mn), corticosterone (eBioscience, TX), GABA (Mybiosource, San Diego, CA), and serotonin (DLD Diagnositika GmbH, Hamburg, Germany) levels were measured using their ELISA kits.

Quantitative polymerase chain reaction (qPCR)

mRNAs (2 μg) from the prefrontal cortex were isolated using a RNeasy Mini kit and their cDNAs were prepared using cDNA synthesis kit (TaKaRa) (Joo et al., 2023). The real time qPCR for serotonin 1A receptor (5-HT1AR), 5-HT1BR, GABA type A receptor subunit alpha1 ($GABA_AR\alpha1$), GABA type A receptor subunit alpha2 ($GABA_AR\alpha2$), and GAPDH rRNAgenes was performed using SYBER premix Ex Taq II (TaKaRa). The thermal cycling condition was as follows: initial denaturation at 95°C for 30 s, denaturation at 95°C for 15 s, annealing at 60°C for 30 s, extension at 72°C for 30 s, and 40 cycling. Gene expression levels were calculated by comparing to GAPDH. Primers are shown in Supplement Table S1.

Immunofluorescence staining

The immunofluorescence staining of brain and colon tissues were performed according to the method of Lee et al. (Lee et al., 2020). Briefly, mice were trans-cardiacally perfused with 4% paraformaldehyde and removed brains and colons. These tissues were post-fixed with 4% paraformaldehyde for 4 h, cytoprotected in 30% sucrose solution, freezed, and sectioned using a cryostat. The sections were incubated for 16 h at 4°C with primary antibodies for GABAARα1, NF-κB, Iba1, and/or CD11c, washed with saline twice, and incubated with secondary antibodies conjugated with Alexa Fluor 488 (1:1,000, Invitrogen) or Alexa Fluor 594 (1:500, Invitrogen). The nuclei were stained with DAPI. Immunostained sections were observed with a confocal laser microscope.

References

Jang, H.M., Lee, K.E., Lee, H.J., Kim, D.H., 2018. Immobilization stress-induced Escherichia coli causes anxiety by inducing NF-κB activation through gut microbiota disturbance. Sci Rep. 8, 13897.

Jang, H.M., Lee, K.E., Kim, D.H., 2019. The Preventive and Curative Effects of Lactobacillus reuteri NK33 and Bifidobacterium adolescentis NK98 on Immobilization Stress-Induced Anxiety/Depression and Colitis in Mice. Nutrients. 11.

Joo, M.K., Ma, X., Yoo, J.W., Shin, Y.J., Kim, H.J., Kim, D.H., 2023. Patient-derived Enterococcus mundtii and its capsular polysaccharides cause depression through the downregulation of NF-κB-involved serotonin and BDNF expression. Microbes Infect.

25, 105116.
Lee, K.E., Kim, J.K., Han, S.K., Lee, D.Y., Lee, H.J., Yim, S.V., Kim, D.H., 2020. The extracellular vesicle of gut microbial Paenalcaligenes hominis is a risk factor for vagus nerve-mediated cognitive impairment. Microbiome. 8, 107.
Ma, Y., Ma, H., Eun, J.S., Nam, S.Y., Kim, Y.B., Hong, J.T., Lee, M.K., Oh, K.W., 2009. Methanol extract of Longanae Arillus augments pentobarbital-induced sleep behaviors through the modification of GABAergic systems. J Ethnopharmacol. 122, 245-50.
